# Billion‐Scale Expansion of Functional hiPSC‐Derived Cardiomyocytes in Bioreactors Through Oxygen Control and Continuous Wnt Activation

**DOI:** 10.1002/advs.202410510

**Published:** 2025-01-23

**Authors:** Pedro Vicente, Lara R. Inocêncio, Asier Ullate‐Agote, Ana F. Louro, João Jacinto, Beatriz Gamelas, Olalla Iglesias‐García, Patxi San Martin‐Uriz, Paula Aguirre‐Ruiz, Gonzalo R. Ríos‐Muñoz, María Eugenia Fernández‐Santos, Alain van Mil, Joost P. G. Sluijter, Felipe Prósper, Manuel M Mazo Vega, Paula M. Alves, Margarida Serra

**Affiliations:** ^1^ iBET Instituto de Biologia Experimental e Tecnológica Apartado 12 Oeiras 2780901 Portugal; ^2^ Instituto de Tecnologia Química e Biológica António Xavier Universidade de Nova de Lisboa Av. da República Oeiras 2780157 Portugal; ^3^ Program of Biomedical Engineering Technological Innovation Division CIMA Universidad de Navarra, and Instituto de Investigación Sanitaria de Navarra (IdiSNA) Pamplona 31008 Spain; ^4^ Bioengineering Department Universidad Carlos III de Madrid Madrid 28911 Spain; ^5^ Department of Cardiology Gregorio Marañón Health Research Institute (IiSGM) Hospital General Universitario Gregorio Marañón Madrid 28007 Spain; ^6^ Centro de Investigación Biomédica en Red de Enfermedades Cardiovasculares (CIBERCV) Instituto Carlos III Madrid 28029 Spain; ^7^ Regenerative Medicine Center Utrecht Laboratory of Experimental Cardiology, Circulatory Health Research Center, University Utrecht University Medical Center Utrecht University Utrecht Utrecht 3508 GA The Netherlands; ^8^ Hematology and Cell Therapy Clínica Universidad de Navarra Pamplona 31008 Spain; ^9^ Hemato‐oncology Program Cancer Division CIMA Universidad de Navarra, and Instituto de Investigación Sanitaria de Navarra (IdiSNA) Pamplona 31008 Spain; ^10^ Centro de Investigación Biomédica en Red Cáncer (CIBERONC) Madrid 28029 Spain

**Keywords:** 3D cell culture, bioprocess scale‐up, hiPSC‐CM expansion, mild hypoxia, stirred‐tank bioreactor, suspension culture

## Abstract

Generation of upscaled quantities of human‐induced pluripotent stem cell‐derived cardiomyocytes (hiPSC‐CM), for therapeutic or testing applications, is both expensive and time‐consuming. Herein, a scalable bioprocess for hiPSC‐CM expansion in stirred‐tank bioreactors (STB) is developed. By combining the continuous activation of the Wnt pathway, through perfusion of CHIR99021, within a mild hypoxia environment, the expansion of hiPSC‐CM as aggregates is maximized, reaching 4 billion of pure hiPSC‐CM in 2L STB. In particular, the importance of i) controlling the dissolved oxygen at 10% O_2_ to reduce reactive oxygen species production and upregulate genes involved in cell proliferation, resulting in higher expansion rates (tenfold) compared to normoxic conditions, and ii) maintaining constant power input per volume as a scale‐up criteria is demonstrated. After expansion, hiPSC‐CM further mature in culture, revealing more mature transcriptional signatures, higher sarcomere alignment and improved calcium handling. This new bioprocess opens the door to time‐ and cost‐effective generation of hiPSC‐CM.

## Graphical abstract

This work shows the development of a bioreactor‐based bioprocess for the expansion of human‐induced pluripotent stem cell‐derived cardiomyocytes (hiPSC‐CM), obtained after cardiac differentiation of hiPSC. By combining the activation of the Wnt pathway, through continuous perfusion of CHIR99021, with a controlled mild hypoxia environment, we maximize the expansion of hiPSC‐CM as aggregates, reaching 4 billion pure and functional hiPSC‐CM in a 2L single‐use bioreactor.

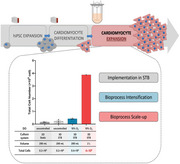

## 1 Introduction

Myocardial infarction (MI) is a leading cause of death worldwide, resulting in the loss of up to one billion cardiomyocytes (CM) due to inadequate oxygenation, nutrient supply, and/or ischemia‐reperfusion injury.^[^ ^1^ ^]^ This leads to persistent complications such as arrhythmias or lower functional performance, leading to a final state of heart failure, with a 5‐year survival rate ≈50%. The limited regenerative capacity of the adult human heart intensifies the burden of MI, emphasizing the need for innovative therapeutic approaches to restore lost cell numbers.^[^ ^2^ ^]^

Human induced pluripotent stem cells (hiPSC) can offer a promising opportunity for generating an unlimited supply of cardiomyocytes (hiPSC‐CM) to restore myocardial tissue post‐MI.^[^ ^2^, ^3^ ^]^ Despite the progress made in the last two decades on the establishment of protocols for efficient expansion and differentiation of hiPSC into hiPSC‐CM,^[^ ^4^, ^5^ ^]^ challenges persist in achieving therapeutic cell numbers.^[^ ^6^, ^7^ ^]^ Most studies have been focused on scaling‐up the initial stages of the hiPSC‐CM process workflow,^[^ ^8^, ^9^ ^]^ since the proliferative capacity of hiPSC‐CM gradually decreases along differentiation.^[^ ^5^, ^10^ ^]^ This restricted ability of hiPSC‐CM has encouraged considerable interest in developing new methodologies to induce proliferation by stimulating cell cycle activity.^[^ ^11^, ^12^, ^13^ ^]^ Buikema and colleagues showed through the synergistic activation of Wnt signalling and low cell density that, GSK‐3β inhibition induced proliferation and hampered maturation of hiPSC‐CM.^[^ ^11^ ^]^ In other studies, researchers have found that exposing CMs to low (10–15%) oxygen concentrations can effectively prolong their immature phenotype and enhance their proliferative capacity in vivo.^[^ ^14^, ^15^ ^]^ Specifically, this mild hypoxic environment protects CMs from premature cell cycle exit, while higher oxygen levels lead to reactive oxygen species (ROS) production and oxidative DNA damage, resulting in cell cycle arrest.^[^ ^15^ ^]^ It remains essential, however, to ensure an efficient control of oxygen concentration in culture as extremely low levels (<10%) can also increase oxidative DNA damage of cardiomyocytes.^[^ ^14^, ^15^ ^]^ All these investigations to stimulate cell cycle activity and proliferation were performed in conventional 2D monolayer static culture systems, which lack the capacity to precisely control key environmental factors, including the dissolved oxygen (DO), and present limited scalability. The scale‐out of 2D static cultures to achieve the numbers of hiPSC‐CM necessary for therapeutic purposes proves inefficient due to the need for large facilities and the high labour costs associated to culturing numerous plates/T‐flasks/cell factories in parallel.^[^ ^16^, ^17^ ^]^ Stirred‐tank bioreactor (STB) systems have the potential to meet these demands.^[^ ^9^, ^18^, ^19^ ^]^ These systems are easy to scale‐up, more cost effective, require less space, less labour‐intensive and are equipped with the appropriate process‐monitoring tools for precise control of critical process parameters that impact on cell production and quality attributes.

In this work, we developed a scalable bioprocess for expansion of hiPSC‐CM as 3D aggregates in STB. By combining the activation of the Wnt pathway, through continuous perfusion of CHIR99021 (CHIR), with precise control of DO at 10%, we maximized the expansion of hiPSC‐CM reaching up to 4 billion functional hiPSC‐CM in a 2L single‐use STB.

## 2 Results

### 2.1 Wnt Pathway Activation Promotes hiPSC‐CM Proliferation in STB

To determine if Wnt pathway activation can stimulate hiPSC‐CM expansion as 3D cell aggregates, we performed a preliminary experiment comparing hiPSC‐CM growth with (CHIR) and without CHIR (w/o CHIR) supplementation in the culture medium (Figure S1, Supporting Information). The data show that CHIR supplementation induced greater hiPSC‐CM expansion (Figure S1A, Supporting Information) that resulted in aggregates with higher diameter (Figure S1B,C, Supporting Information) than no‐CHIR condition, confirming the role of CHIR in supporting cell proliferation in 3D cell culture

**Figure 1:**
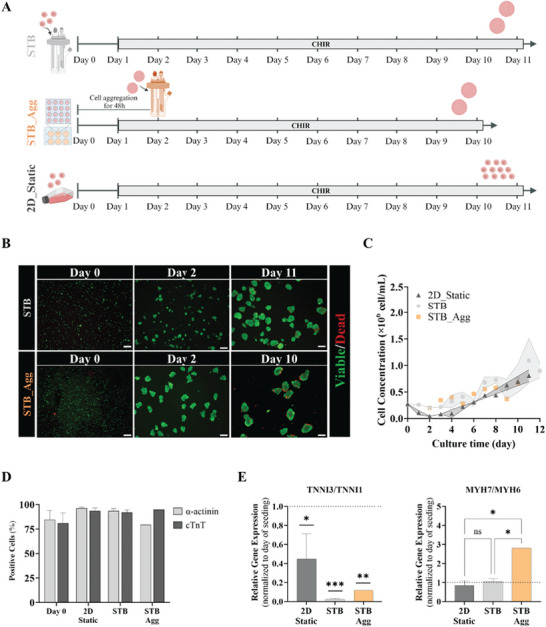
Wnt pathway activation promotes hiPSC‐CM proliferation in STB. A) Schematic illustration of the expansion protocol for human induced pluripotent stem cell‐derived cardiomyocytes (hiPSC‐CM). The protocol involves Wnt‐signalling activation through the addition of CHIR99021 (CHIR). Three distinct strategies are depicted: monolayer culture (2D_Static) and suspension culture in stirred‐tank bioreactors (STB) under different inoculation strategies (single cells – STB, and aggregates – STB_Agg). B) Fluorescence images of cell aggregates in the suspension culture conditions for STB at days 0, 2, 11, and for STB_Agg at days 0, 2, 10, stained with fluorescein diacetate (FDA, live cells, green) and Propidium iodide (PI, dead cells, red). Scale bar = 200 µm. C) Viable cell growth profile throughout hiPSC‐CM expansion. D) Flow cytometry analysis of cardiomyocyte‐specific markers *α*‐actinin and cardiac troponin T (cTnT), performed at inoculum (day 0) and at the last day of hiPSC‐CM expansion. E) Ratios of the relative gene expression of cardiomyocyte maturation genes. TNNI3 to TNNI1 and MYH7 to MYH6. Data are mean ± SEM. *p* ≤ 0.05(*), *p* ≤ 0.01 (**), *p* ≤ 0.001 (***) and n.s. (p > 0.05) by one‐way ANOVA with Tukey's post hoc multiple‐comparisons test. n_2D_Static_ = 3, n_STB_ = 3, n_STB_Agg_ = 1.

We then evaluated two inoculation strategies of hiPSC‐CM in STB, i.e., single cells (STB) versus aggregates (STB_Agg), and compared cell growth profile and phenotype, with cell expansion in the 2D monolayer static systems (2D_Static) previously reported^[^ ^11^ ^]^ (**Figure** **1**A). For all conditions we used hiPSC‐CM that were differentiated from hiPSC using a small molecule‐based Wnt‐modulation protocol described in the literature.^[^ ^20^ ^]^ After confirming that the used hiPSC lines were positive for pluripotency markers SSEA‐4 and TRA‐1‐60, and negative for the early differentiation marker SSEA‐1 (Figure S2A, Supporting Information) by flow cytometry analysis, we induced CM differentiation (Figure S2B, Supporting Information). The differentiating cells displayed expression of pluripotency genes in the early stages (*NANOG*, *POU5F1*, Figure S2C, Supporting Information), followed by the expression of cardiac progenitor marker from day 5 (*NKX2.5*, *GATA4*, Figure S2D, Supporting Information) before committing to the cardiomyogenic lineage (Figure S2E,F, Supporting Information). By day 11, when more than 80% of cells were beating (Movie S1, Supporting Information), hiPSC‐CM were dissociated and inoculated as single cells at a density of 0.27 × 10^6^ mL^−1^ either on STB (Figure 1A) or T‐flasks (2D_Static, Figure 1A). In addition, hiPSC‐CM were also plated in AggreWell plates to induce the formation of aggregates, which were subsequently transferred to STB after two days of formation (STB_Agg, Figure 1A).

Expansion of hiPSC‐CM was stimulated by the addition of CHIR to the culture media. In both STB cultures, hiPSC‐CM were able to proliferate as 3D aggregates, displaying high cell viability (Figure 1B), increased aggregate size over time (Figure S3A, Supporting Information) and demonstrating spontaneous beating activity (Movies S2–S4, Supporting Information). The hiPSC‐CM growth profile and expansion factor in STB was similar to that observed in 2D_Static, reaching a maximum concentration of 1.0 × 10^6^ cell mL^−1^ and viability on day 11 (Figure 1C; Figure S3B and Table S1, Supporting Information), contrasting with STB_Agg condition, where the peak of cell concentration was lower (0.6 × 10^6^ cell mL^−1^) and attained earlier (day 10, Figure 1C). These observations confirm that the inoculation strategy impacts hiPSC‐CM expansion, which was further confirmed by flow cytometry analysis, which revealed that CHIR treatment slightly enriched CM purity (Figure 1D), with a higher percentage of cells positive for cardiac markers after hiPSC‐CM expansion in 2D_Static and STB conditions (90% at day 11 vs 80% at day 0). Real‐time quantitative PCR analysis showed a decreased *TNNI3* to *TNNI1* ratio and *MYH7* to *MYH6* ratio in the STB condition compared to STB_Agg, thereby suggesting a higher maturation arrest phenotype at the transcriptional level in the STB strategy on day 11 day (Figure 1E).

### 2.2 Dissolved Oxygen Controlled at 10% O_2_ Reduces ROS and Improves hiPSC‐CM Proliferation in STB

**Figure 2:**
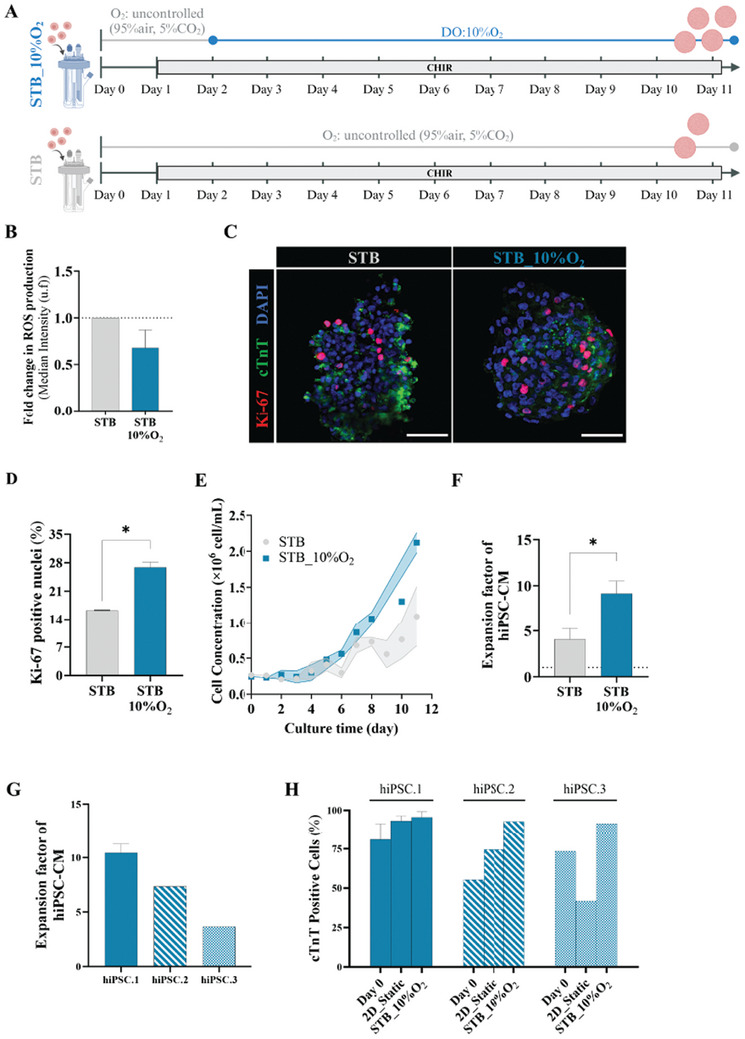
Mild hypoxia (10% O_2_) promotes hiPSC‐CM proliferation in stirred‐tank bioreactors. A) Schematic illustration of the expansion protocol for human induced pluripotent stem cell‐derived cardiomyocytes (hiPSC‐CM) in STB. Two distinct strategies under different oxygen conditions are depicted (STB and STB_10%O_2_). B) Flow cytometry analysis of reactive oxygen species (ROS) production fold change, performed at day 8 of hiPSC‐CM expansion and normalized to STB condition. C) Immunofluorescence images of cell proliferation marker Ki‐67 (Ki‐67, red) and of cardiac troponin T (cTnT, green). Nuclei were counterstained with DAPI (blue), for the STB and for the STB_10%O_2_ conditions. Scale bar = 200 µm. D) Quantification of the percentages of Ki‐67 positive cells from the immunofluorescence images shown in (C) and equivalents (*n* = 3). E) Viable cell growth profile throughout hiPSC‐CM expansion. Data for STB corresponds to the data in Figure 1C. F) Fold increase in viable hiPSC‐CM concentration at end of expansion (day 11), compared to the initial inoculum. G) Fold increase in hiPSC_CM concentration positive for cTnT cardiac marker at end of expansion (day 11), compared to the initial inoculum for three distinct cell lines, hiPSC.1, hiPSC.2 and hiPSC.3. H) Flow cytometry analysis of CM‐specific cTnT, performed at inoculum (day 0) and at the last day of hiPSC‐CM expansion (day 11) for three distinct cell lines. Error bars represent SEM, *, *p* ≤ 0.05. n_hiPSC.1_ = 3, n_hiPSC.2_ = 1, n_hiPSC.3_ = 1) by paired t test.

We subsequently asked whether a mild hypoxic environment would impact on hiPSC‐CM culture in STB (**Figure** **2**A). Online monitoring of DO confirm that in the STB condition, DO ranged from 80% to 100% of oxygen concentration in air, saturated (Figure S4A, Supporting Information), whereas in STB_10%O_2_, DO maintained a constant DO level at 47.6% of oxygen concentration in air, saturated (corresponding to 10% oxygen), after day 2 (Figure S4B, Supporting Information). We observed a decrease in reactive oxygen species (ROS) production (≈0.7‐fold, Figure 2B) together with a significant increase in cell cycle activity, measured by the cell cycle marker Ki‐67, when hiPSC‐CM were cultured in STB_10%O_2_ condition compared to atmospheric normoxic condition STB (Figure 2C,D). Indeed, our results showed that the mild hypoxic condition improved the expansion of hiPSC‐CM in 3D aggregates in STB by twofold, yielding 2.1 × 10^6^ viable cell mL^−1^ at day 11 (Figure 2E), hereby corresponding to an expansion factor of 9.2 ± 1.4 (Figure 2F; Table S1, Supporting Information), and demonstrated spontaneous beating activity (Movie S5, Supporting Information). Importantly, expansion capacity in STB_10%O_2_ was reproducible for CMs derived from three independent hiPSC lines isolated from different donors and tissues (hiPSC.1, hiPSC.2, hiPSC.3). Although some differences in hiPSC‐CM expansion factors were attained among the three hiPSC lines (Figure 2G), CHIR treatment combined with low oxygen levels in STB consistently displayed an improvement in CM purity, measured by flow cytometry analysis of cTnT positive cells (Figure 2H), and being higher when compared to cell expansion in 2D static culture under normoxic conditions (Figure 2H).

Finally, our data also demonstrated the importance of controlling the DO at 10% O_2_ to maximize cell expansion factors; expanding hiPSC‐CM under DO of 5% or 15% O_2_ resulted in higher cell death and aggregate size heterogeneity as well as lower cell expansion capacity and reduced percentage of cells expressing cardiac markers (Figure S5A–D, Supporting Information).

### 2.3 STB Operated at DO of 10% O_2_ Upregulates the Expression of Genes Associated with Hypoxia and Cell Proliferation Pathways

**Figure 3:**
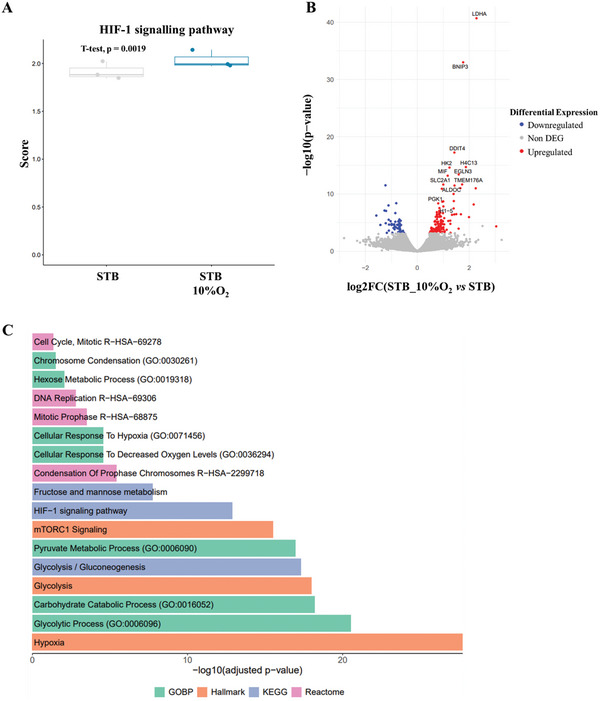
hiPSC‐CM expansion in a mild hypoxic (10% O_2_) environment activates hypoxia, cell metabolism and cell proliferation pathways. A) Boxplot representation of the ssGSEA scores for the hypoxia‐inducible factor 1 (HIF‐1) signaling pathway gene set (hsa04066). The p value was obtained by a paired t test. B) Differentially expressed genes (DEGs) in the comparison of STB and STB_10%O_2_ conditions. In the volcano plot, the x axis indicates log2 fold change (FC), and the y axis indicates statistical significance with the ‐log10(p‐value). Genes with an adjusted p value < 0.05 are considered upregulated (red) if the logFC > 0 and downregulated (blue) if the logFC < 0. Non‐DEGs are shown in grey. C) Barplot of upregulated gene sets when comparing STB_10%O_2_ versus STB in an overrepresentation analysis for the GO biological processes (green), MSigDB Hallmark (orange), KEGG (blue) and Reactome (pink) databases. n_STB_ = 3, n_STB_10%O2_ = 3.

To understand the molecular mechanisms underlying enhanced cell proliferation and cell cycle activity we subsequently analyzed gene expression profiles of hiPSC‐CM cultured in STB_10%O_2_ versus STB. Our findings revealed an upregulation of the hypoxia‐inducible factor (HIF) pathway in STB_10%O_2_, as compared to STB (**Figure** **3**A) and confirms the induction of hypoxic conditions when DO was controlled at 10% O_2_. Analysis of differential gene expression revealed higher expression of genes involved in hypoxia (e.g., *BNIP3*, *DDIT4*, *EGLN3*, *MIF*), glycolysis (e.g., *LDHA*, *HK2*, *ALDOC*, *SLC2A1*, *PGK1*), and cell proliferation (e.g., *H4C13*, *TMEM176A*, *H1‐5*) in hiPSC‐CM cultured in STB_10%O_2_ compared to those in STB (Figure 3B). Our results also demonstrated that the biological processes and pathways upregulated in STB_10%O_2_, were related to hypoxia, cell metabolism, protein synthesis and cell growth and proliferation in hiPSC‐CM (Figure 3C).

**Figure 4:**
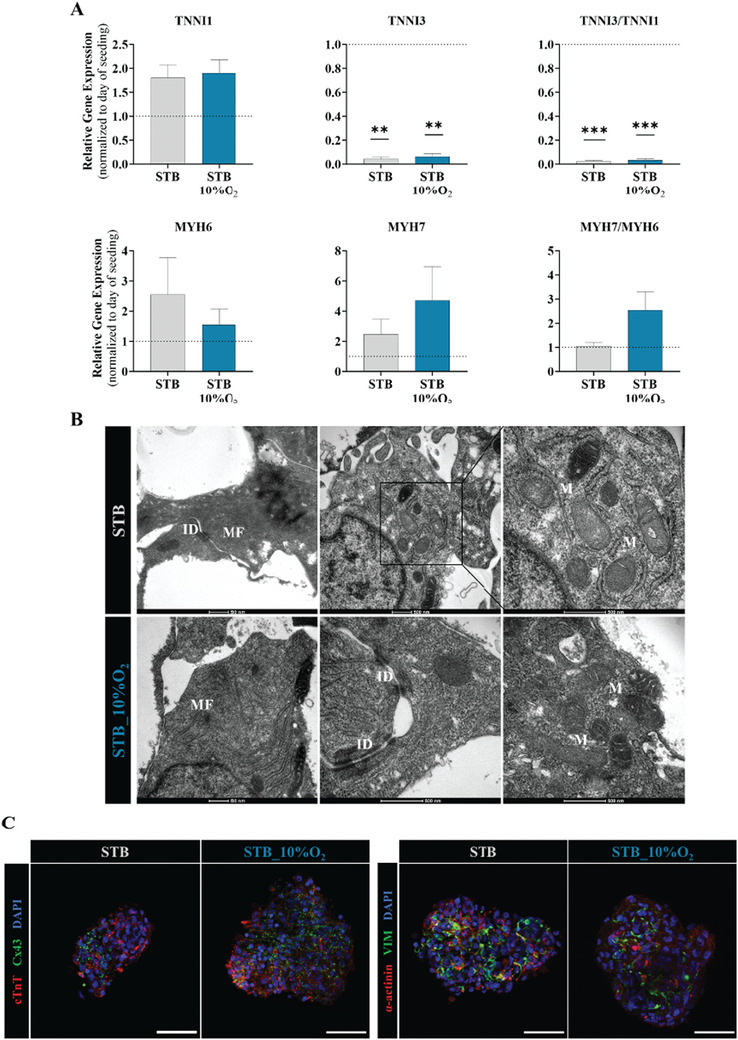
Phenotypic characterization of hiPSC‐CM after 11 days of expansion. A) Relative gene expression of CM maturation genes and ratios, TNNI3 to TNNI1, and MYH7 to MYH6 compared to STB inoculum. B) Transmission electron microscopy images of hiPSC‐CM aggregates at day 8 of expansion for the STB and STB_10%O_2_. Myofibrils (MF), intercalated disks (ID) connecting adjacent CMs, and mitochondria (M). Scale bars = 500 nm. C) Representative immunofluorescence staining of cardiac troponin T (cTnT, red), connexin‐43 (Cx43, green), and of *α*‐actinin (red), vimentin (VIM, green) of hiPSC‐CM aggregates after expansion. Nuclei were counterstained with DAPI (blue). Scale bar = 200 µm. Error bars represent SEM; **, p < 0.01; ***, p < 0.001. n_2D_Cntrl_ = 3, n_STB_ = 3, n_STB_10%O2_ = 3 by paired t test.

Next, we evaluated the phenotype of hiPSC‐CM following expansion in STB exposed to different DO concentrations. Our results confirmed that hiPSC‐CM maintained their immature phenotype in both STB_10%O_2_ and STB. We did not find significant differences in gene expression of cardiac markers (*TNNI1*, *MYH6*, *MYH7*) and in the *MYH7* to *MYH6* ratio after 11 days of expansion, compared to inoculum (day 0, **Figure** **4**A). We observed a statistically significant downregulation of the mature cardiac marker *TNNI3*, with a significant decrease in the *TNNI3* to *TNNI1* ratio at the end of expansion (Figure 4A) in both STB_10%O_2_ and STB. Transmission electron microscopy analysis of hiPSC‐CM aggregates also showed complete sarcomere disassembly, mostly unaligned myofibrils (MF), and mitochondria (M) with few cristae and low contrast in both STB_10%O_2_ and STB (Figure 4B). Immunostaining analysis confirmed the expression of cardiac markers in hiPSC‐CM aggregates, with heterogeneous and underdeveloped distribution of cTnT, connexin‐43 (Cx43), *α*‐actinin, and vimentin (VIM) positive cells in both STB conditions (Figure 4C).

### 2.4 Scale‐up hiPSC‐CM Expansion Process from 0.2L to 2L STB

**Figure 5:**
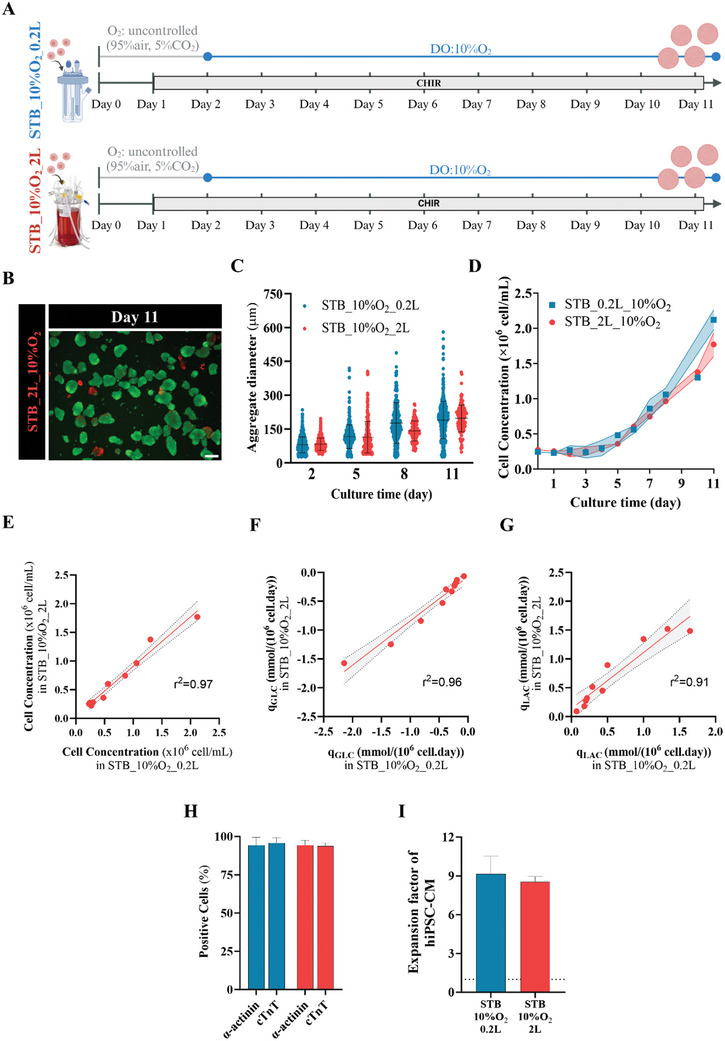
Scale‐up to 2L bioreactors validates the hiPSC‐CM expansion protocol for high‐volume production of hiPSC‐CM aggregates. A) Schematic illustration of the scale‐up of the optimized hiPSC‐CM expansion protocol (STB_10%O_2_) to 2‐liter STB. The two distinct strategies are depicted: STB_10%O_2_ condition in 0.2L STB (STB_10%O_2__0.2L) and in 2‐liter STB (STB_10%O_2__2L). B) Fluorescence images of cell aggregates at days 11, in the STB_10%O_2__2L strategy, stained with fluorescein diacetate (FDA, live cells, green) and propidium iodide (PI, dead cells, red). Scale bar = 200 µm. C) Average hiPSC‐CM aggregate diameter estimated at days 2, 5, 8, 11 in in STB_10%O_2__0.2L and in STB_10%O_2__2L (more than 200 aggregates per condition were analyzed). D) Viable cell growth profile throughout hiPSC‐CM expansion. E–G) Comparison of cell concentration (E), and rates of glucose (GLC) consumption (F) and lactate (LAC) production (G) throughout hiPSC‐CM expansion, between STB_10%O_2__0.2L and STB_10%O_2__2L, with straight line representing the best linear fit to the data (r^2^—Pearson's correlation) and the grey band representing a 95% confidence interval. H) Flow cytometry analysis of CM‐specific markers *α*‐actinin and cTnT, performed at the last day of hiPSC‐CM expansion (day 11). I) Fold increase in hiPSC‐CM concentration, positive for cTnT marker, at end of expansion (day 11), compared to the initial inoculum. Error bars represent SEM. n_STB_10%O2_0.2L_ = 3, n_STB_10%O2_2L_ = 3.

We then explored the feasibility to scale‐up hiPSC‐CM expansion (STB_10%O_2_ strategy) to a 2L single‐use STB (2L Univessel SU), aiming to produce hiPSC‐CM in a clinical‐relevant billion scale (**Figure** **5**A). The scale‐up criteria used was to maintain the power input per volume (P/V) constant across the 0.2L and 2L STB.

Our results demonstrated that hiPSC‐CM successfully aggregated in 2L STB, demonstrating high cell viability (Figure 5B), increased aggregate size over time and demonstrated spontaneous beating activity (Figure 5C; Movie S6, Supporting Information). Importantly, hiPSC‐CM growth profile, cell expansion factor, aggregate size distribution, cell metabolism (the profiles of specific rates of glucose consumption and lactate production), and CM yield (estimated by the percentage of cells expressing cTnT and *α*‐actinin) were very similar across 0.2L and 2L STB cultures (Figure 5C–H). All together, these results confirmed the successful scale‐up of hiPSC‐CM expansion to 2L bioreactors capable of generating ≈4 billion cells per run (Table S1, Supporting Information).

### 2.5 hiPSC‐CM Expanded in STB have the Capacity to Further Mature In Vitro

**Figure 6:**
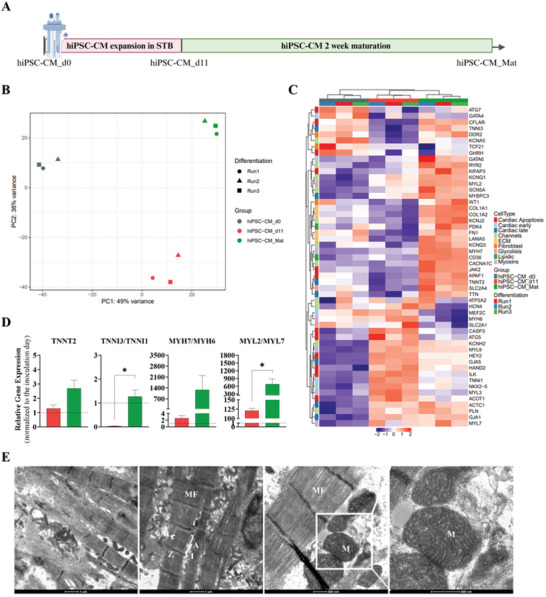
hiPSC‐CM mature in culture after two weeks maturation. A) Schematic illustration of the hiPSC‐CM maturation. B) Principal components analysis of hiPSC‐CM after cardiac differentiation (hiPSC‐CM_d0), expansion (hiPSC‐CM_d11) and after two weeks of maturation (hiPSC‐CM_Mat). The percentage of variance explained by PC1 and PC2 is depicted in the axes. The color code corresponds to the grouping and the shape to the differentiation of origin C) Heatmap representing the scaled expression of cardiac‐specific genes after variance stabilizing transformation (vst). Heatmap rows are color coded by overall cardiac function D) Relative gene expression of cardiomyocyte TNNT2 gene and ratios of TNNI3 to TNNI1, of MYH7 to MYH6, and of MYL2 to MYL7 compared to STB inoculum. E) Transmission electron microscopy images of hiPSC‐CM expanded in STB_10%O_2_ and cultured without CHIR for 21 days in monolayers. Myofibrils (MF), Z‐disks (Z), isotropic‐band of I‐band (I), anisotropic‐band or A‐band (A), H Zone (H), and mitochondria (M). Scale bars = 1 µm and 500 nm. n_hiPSC‐CM_d0_ = 3, n_hiPSC‐CM_d11_ = 3, n_hiPSC‐CM_Mat_ = 3. Error bars represent SEM; *, p < 0.05; by paired t test.

To confirm whether the hiPSC‐CM expanded cells presented the capacity to further mature in culture, we harvested hiPSC‐CM aggregates and enzymatically dissociated them into single cell suspension. We subsequently plated the cells in static culture systems and cultured hiPSC‐CM in standard medium in the absence of CHIR inside incubators with a humidified atmosphere of 5% (v/v) CO_2_ and 95% (v/v) air for two weeks. We performed bulk RNA sequencing to analyze the transcriptomic signatures of hiPSC‐CM at day 0 (hiPSC‐CM_d0), at day 11 (hiPSC‐CM_d11), and after maturation (hiPSC‐CM_Mat, **Figure** **6**A). Strong correlations were found between biological replicates within conditions, suggesting consistency in hiPSC‐CM differentiation, expansion, and maturation. Principal component analysis (PCA) was used to explore broad differences between the three hiPSC‐CM groups (Figure 6B). The results revealed a clear clustering pattern, with each independent replicate grouping together within their respective group. Principal Component 1 (PC1), accounting for 49% of the total variance, effectively separates hiPSC‐CM_d0 from hiPSC‐CM_d11, as well as from hiPSC‐CM_Mat. Meanwhile, Principal Component 2 (PC2), explaining 38% of the variance, primarily distinguishes hiPSC‐CM_d11 from hiPSC‐CM_Mat (Figure 6B).

Furthermore, global RNA expression for genes strongly expressed in the heart, differed across the different groups. In general, a higher expression of genes associated with cardiac function, ion channels, extracellular matrix and mature‐related myosin's can be observed in hiPSC‐CM upon maturation (Figure 6C). Analysis by Real‐time quantitative PCR confirmed a higher expression of the adult related genes (*TNNI3*, *MYH7*, *MYL2*), together with similar expression of *TNNI1*, *TNNT2*, *MYH6* (Figure 6D; Figure S6A, Supporting Information). This corresponded to increased ratios of *TNNI3* to *TNNI1*, *MYH7* to MYH6, and *MYL2* to *MYL7*, indicative of enhanced maturation (Figure 6D). Upon analysing differentially expressed genes patterns, we identified four distinct groups (Figure S6B, Supporting Information). hiPSC‐CM_Mat exhibited the lowest expression for genes in group 1 and the highest expression for genes in group 4 (Figure S6B, Supporting Information). In contrast, hiPSC‐CM_d11 showed the lowest expression for genes in group 2 and the highest expression for genes in group 3 (Figure S6B, Supporting Information).

Specifically, examining the top 10 GO biological processes for each group, we observed processes involved in heart development in group 1, which aligns with differentiation toward a cardiac lineage and further confirms the higher expression in hiPSC‐CM_d0 (Figure S6C, Supporting Information). In group 2, we retrieved processes related to extracellular matrix organization, which is consistent with the lower expression in hiPSC‐CM_d11 due to their immature phenotype (Figure S6C, Supporting Information). Group 3 showed processes involving gene expression, translation, biogenesis and cellular respiration potentially supporting the higher proliferative capacity of hiPSC‐CM_d11 (Figure S6C, Supporting Information). Group 4 included processes linked to cardiac muscle development, contraction, and myofibril assembly, further supporting the more mature phenotype of hiPSC‐CM_Mat (Figure S6C,D, Supporting Information). Additionally, we identified several genes associated with cardiac function that were differentially expressed between the four groups (e.g., *ACTC1*, *MYBPC3*, *PLN*), with maturation genes such as *MYL2* and *MYH7* being more expressed in group 4 (Figure S6E, Supporting Information).

TEM analysis revealed a highly organized microstructure of hiPSC‐CM upon maturation, characterized by well‐aligned sarcomeres exhibiting discernible Z‐line, H‐zone, and I‐ and A‐bands and with evident H zones (H) (Figure 6E). Furthermore, an abundance of mitochondria, featuring well‐defined cristae, was observed particularly in proximity to myofibers (Figure 6E). The high mitochondrial density is consistent with an increased energetic workload required for muscle contraction, likely due to a shift from glycolytic to oxidative metabolism.

**Figure 7:**
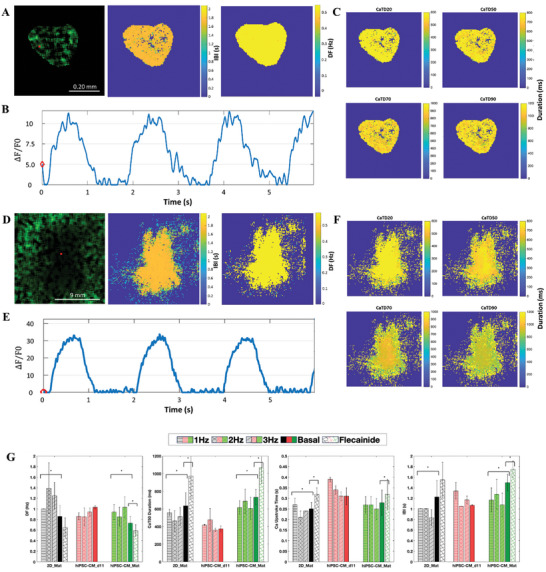
Functional characterization of hiPSC‐CM after two weeks of maturation with comparisons to 2D mature culture conditions. A) Calcium transient optical mapping signal for 3D culture. Left: raw fluorescence frame. Middle: inter‐beat interval (IBI) map. Right: Dominant frequency (DF) map. B) Activation profile of three complete calcium transients’ activations for the red pixel in (A). C) Temporal analysis maps for calcium transient duration (CaTD) at various repolarization values (20%, 50%, 70%, and 90%) for the 2D culture. D) Calcium transient optical mapping signal for a 2D culture. Left: raw fluorescence frame. Middle: inter‐beat interval (IBI) map. Right: dominant frequency (DF) map. E) Activation profile of three complete calcium transient activations from the red pixel shown in (D). F) Temporal analysis maps for calcium transient duration (CaTD) at various repolarization values (20%, 50%, 70%, and 90%). G) Quantitative analysis of dominant frequency, CaTD50, IBI, and calcium depolarization upstroke duration across experimental groups, including 2D culture (2D_Mat), 3D immature (hiPSC‐CM_d11), and 3D mature (hiPSC‐CM_Mat) conditions, under basal conditions, different stimulation frequencies (1–3 Hz), and post‐flecainide antiarrhythmic drug administration. * Indicates statistical significance (p < 0.05). Data are presented as mean ± standard deviation. Abbreviations: DF, dominant frequency; CaTD, calcium transient duration; IBI, inter‐beat interval.

Calcium handling in 2D mature cultures (2D_Mat), hiPSC‐CM_d11, and hiPSC‐CM_Mat aggregates was assessed using a fluorescent calcium indicator to detect spontaneous calcium transients during each cardiac contraction cycle (**Figure** **7**A–F). Analysis of these transients allowed for the estimation of calcium kinetics parameters and a more comprehensive comparison between 2D and 3D culture conditions. A global map analysis of 2D_Mat and hiPSC‐CM_Mat was used to calculate the dominant frequency (DF, Figure 7A,D), inter‐beat interval (IBI), and calcium transient repolarization values (CaTD20, CaTD50, CaTD70, and CaTD90; Figure 7A,D for 3D and 2D conditions respectively).

At baseline, 2D_Mat exhibited stable calcium dynamics with a DF of 0.86 ± 0.20 Hz, a CaTD50 duration of 634.53 ± 147.63 ms, and a calcium upstroke time of 0.25 ± 0.03 ms (Figure 7D–G). Additionally, no arrhythmic propagation patterns, such as reentries or fast locally self‐triggered scattered activity, were observed in the 2D culture (Figure S7, Movie S7, and Tables S2–S4, Supporting Information). A quantitative comparison of the electrophysiological and calcium handling parameters across all experimental conditions is detailed in Tables S3 and S4, Supporting Information. Compared to 2D_Mat, hiPSC‐CM_d11 aggregates displayed higher DF (1.03 ± 0.03 Hz vs 0.86 ± 0.20 Hz; p = 0.2375), faster IBI (1.07 ± 0.01 s vs 1.22 ± 0.31 s; p = 0.0224), shorter CaTD50 duration (373.12 ± 33.46 ms vs 634.52 ± 147.63 ms; p = 0.0302), and longer calcium upstroke time (0.31 ± 0.04 ms vs 0.25 ± 0.03 ms; p = 0.0714), reflecting their immature state (Figure 7G; Tables S3 and S4, Supporting Information). Meanwhile, hiPSC‐CM_Mat aggregates (Movie S8, Supporting Information) exhibited metrics closer to 2D_Mat cultures, with a slower DF of 0.73 ± 0.13 Hz (p = 0.1171), longer IBI of 1.50 ± 0.16 s; p = 0.0003), a CaTD50 duration of 733.73 ± 84.32 ms (p = 0.0819), and a similar calcium upstroke time of 0.28 ± 0.06 ms (p = 0.8285, Figure 7G; Tables S3 and S4, Supporting Information). hiPSC‐CM_Mat aggregates show a more efficient excitation‐contraction coupling (ECC) apparatus compared to hiPSC‐CM_d11, allowing for quicker sarcolemma depolarization, faster sarcoplasmic reticulum (SR) calcium sequestration, and rapid sarcolemma repolarization. Additionally, hiPSC‐CM_Mat aggregates exhibited improved antiarrhythmic properties, including lower dominant frequency (DF) and longer inter‐ IBIs and CaTD durations, indicating reduced susceptibility to arrhythmogenic behavior when compared to hiPSC‐CM_d11 (Figure 7G, Tables S3 and S4, Supporting Information). Furthermore, analysis by real‐time quantitative PCR confirmed a higher expression, although not statistically significant, of the adult related genes (*TNNI3* and *MYH7*), and increased ratios of *TNNI3* to *TNNI1* and *MYH7* to MYH6 in hiPSC‐CM_Mat when compared to the 2D_Mat condition (Figure S8, Supporting Information). These results indicate that the hiPSC‐CM expanded in STB present improved functionality and gene expression after maturation compared to the hiPSC‐CM expanded in 2D static conditions.

When treated with the antiarrhythmic agent flecainide, a sodium channel blocker, no significant differences in DF, CaTD50, or calcium upstroke time were observed between 2D_Mat and hiPSC‐CM_Mat (Figure 7G). Additionally, in 2D mature cultures, conduction velocity (CV) was also assessed (Figure S9, Supporting Information). Results showed a significant reduction in CV after flecainide treatment for all tested rhythms (p<0.05 for all of them), consistent with its known electrophysiological effects (Figure S9, Supporting Information). Under rapid stimulation at 3 Hz, hiPSC‐CM_Mat aggregates tolerated this rapid electrical stimulation without failing, while hiPSC‐CM_d11 aggregates entered a refractory state, further highlighting the functional maturity and improved electrophysiological stability of the hiPSC‐CM_Mat aggregates (Figure 7G, Tables S3 and S4, Supporting Information). These results demonstrate that hiPSC‐CM_Mat aggregates have better‐developed ion channel function, more efficient calcium handling, and greater overall cell maturity (Figure 7G).

## 3 Discussion

In recent years, significant progress has been made in the development of cell‐based therapies targeting the remuscularization of the injured myocardium post‐MI, with particular attention to the therapeutic potential of transplanting hiPSC‐CM.^[^ ^21^, ^22^ ^]^ However, achieving production of over 1 billion clinically relevant hiPSC‐CM in a single run remains challenging. In this study, we developed a bioreactor‐based bioprocess for the expansion of hiPSC‐CM, obtained after cardiac differentiation of hiPSC that ensures the production of high numbers of pure and functional cells in a scalable, cost‐effective, and reproducible manner.

While cardiac differentiation protocols have been continuously improved over the past decade, these protocols still present some limitations. including the low scalability, high costs, and high variability across hiPSC lines.^[^ ^9^, ^19^, ^23^, ^24^, ^25^, ^26^ ^]^ The recent establishment of bioreactor‐based protocols as scalable culture systems for hiPSC‐CM production still rely on directed differentiation of hiPSC into CM in suspension, as 3D aggregates^[^ ^9^, ^19^ ^]^ or on microcarriers.^[^ ^27^ ^]^ Although they represent a significant improvement compared to conventional static 2D monolayer culture systems regarding scalability and cell yields,^[^ ^25^ ^]^ they are still inefficient for application in cell therapy at a larger scale, with low differentiation yields reported (3 CM are generated per hiPSC).^[^ ^9^, ^19^, ^25^ ^]^ To address these challenges, we developed a scalable bioprocess in STB that promotes the expansion of hiPSC‐CM and maximizes the global differentiation yield by over tenfold, i.e., 30 hiPSC‐CM can be generated per hiPSC. This was achieved by culturing 3D hiPSC‐CM aggregates for an additional 11 days in STB with continuous addition of CHIR, through perfusion, in a controlled mild hypoxic environment. Given that reagents costs for hiPSC expansion and cardiomyocyte differentiation are the major expense in our process, extending the culture period for hiPSC‐CM aggregate expansion by 11 days allows us to reduce costs by up to 80% compared to the conventional protocol only including hiPSC expansion and differentiation process to achieve an equivalent hiPSC‐CM yield

The effectiveness of CHIR as a potent CM mitogen and the influence of oxygen levels on CM cell cycle activity have been previously evaluated in hiPSC‐CM cultured in 2D monolayer systems.^[^ ^11^, ^12^, ^13^, ^14^, ^15^ ^]^ However, the combined stimulation of these biological factors and their fine control in STB has never been reported. We demonstrated the importance of controlling the DO at 10% O_2_ to reduce ROS production and enhance cell cycle activity, ultimately resulting in higher cell expansion factors when compared to hiPSC‐CM expanded in atmospheric normoxic conditions without DO control. In particular, expanding hiPSC‐CM under 5% or 15% DO resulted in higher aggregate death and lower cell expansion, compared to hiPSC‐CM cultured at 10% DO. This is likely due to the high DNA damage and increased cell cycle arrest, as reported in both low‐oxygen and oxygen‐rich environments.^[^ ^14^, ^15^ ^]^ These findings highlight the importance of using culture systems like STB, which have the appropriate process‐monitoring tools for precise control of critical process parameters, such as DO, to ensure optimal cell production and cell quality attributes.

After hiPSC‐CM expansion in mild hypoxia, a higher CM enrichment was achieved, which resulted from a more focused expansion toward myocytes, as previously reported.^[^ ^11^ ^]^ This effect was observed in all 3 hiPSC lines evaluated (>90% of cTnT‐positive cells), and particularly pronounced in hiPSC.2 and hiPSC.3, which exhibited lower CM differentiation efficiencies compared to the hiPSC.1. The scalable bioprocess herein designed for hiPSC‐CM expansion consistently enriches CM content, reducing the need for additional purification strategies that traditionally rely on lactate‐based purification^[^ ^28^, ^29^ ^]^ fluorescence‐ (FACS), or magnetic‐ activated cell sorting (MACS) techniques.^[^ ^17^, ^30^, ^31^ ^]^

Previous studies showed the importance of disrupting the cell‐cell contact to enhance CM proliferation.^[^ ^11^, ^32^, ^33^ ^]^ It is plausible that hiPSC‐CM cultured in STB and inoculated as 3D aggregates, with extensive cell‐cell contact, may undergo cell cycle arrest thus hindering cell proliferation and promoting maturation, when compared to single cell inoculation. Another important factor impacting hiPSC‐CM is the aggregate diameter. It is well accepted that, if the aggregate diameter exceeds 300 µm, their centres can become necrotic due to insufficient oxygen and nutrient supply.^[^ ^34^, ^35^, ^36^ ^]^ In our study, we observed high cell death when aggregate size reached 300 µm, a value only achieved in the STB_Agg strategy, thus potentially justifying the lower expansion capacity of this approach compared to the STB one.

One of the most critical parameters for bioprocess scale‐up is to maintain P/V constant across different STB scales, thus ensuring consistent cell culture homogenization, liquid‐gas transfer, and shear stress.^[^ ^37^ ^]^ In our study, using such criteria, we were able to scale‐up the hiPSC‐CM expansion process from 0.2L to 2L without impacting expansion factor, growth and metabolic profiles, and CM purity. Importantly, this strategy allowed the generation of ≈4 billion hiPSC‐CM in a single 2L STB, thus fulfilling the clinical need of a patient undergoing a potential regenerative therapy,^[^ ^3^ ^]^ underscoring its potential for large‐scale production.

One major concern in the transplantation of immature cells is their potential to cause arrhythmias.^[^ ^38^ ^]^ The hiPSC‐CM obtained after expansion in STB still present an immature phenotype, which can be reverted to a more matured state by withdrawing CHIR and oxygen control. This led to improved sarcomere organization, expression of heart‐specific genes, electrophysiological response, and force generation. Additional approaches can be easily integrated in our bioprocess to further improve the structural and functional maturation of hiPSC‐CM, including 3D tissue engineering,^[^ ^39^, ^40^ ^]^ electrical and mechanical stimulation,^[^ ^39^, ^40^ ^]^ hormonal treatments,^[^ ^41^, ^42^, ^43^ ^]^ co‐culture with non‐myocyte cells^[^ ^44^, ^45^ ^]^ and metabolic‐based approaches.^[^ ^46^, ^47^ ^]^

Previous research has shown that in 2D monolayer static cultures, hiPSC‐CM can undergo up to five passages before their proliferative potential is lost;^[^ ^11^ ^]^ at STB scale this was not evaluated. Therefore, further investigations should focus on assessing the impact of serial cell passages on the continuous expansion of hiPSC‐CM in STB. In the future, other cardiac differentiation protocols could be used to generate hiPSC‐CM before the expansion in STB. Here, we used the B27‐based differentiation,^[^ ^5^ ^]^ but Heparin^[^ ^48^ ^]^ and CDM3^[^ ^10^ ^]^ protocols have also successfully generated hiPSC‐CM for expansion in monolayer static systems.^[^ ^20^ ^]^ Additionally, we can integrate the hiPSC expansion and CM differentiation as 3D cell aggregates into our hiPSC‐CM expansion process to streamline hiPSC‐CM production in STB. However, as we reported, if the aggregate diameter exceeds 300 µm, their centres can become necrotic. Therefore, efficient methods for dissociating hiPSC‐CM aggregates or to reduce aggregate size before inducing their expansion would be required.

## 4 Experimental Section

### hiPSC Expansion

Three hiPSC lines were used for this study (Table S5, Supporting Information). Cells were routinely expanded using hiPSC culture media (Table S6, Supporting Information) on Matrigel‐coated static culture systems (e.g., T‐flasks) in a humidified atmosphere at 37 °C and 5% (v/v) CO_2_ and 95% (v/v) air. In brief, hiPSC were passaged every 3–4 days, and at 80–90% confluency cells were dissociated using Versene (Gibco, Thermo Fisher Scientific) for 20 min at 37 °C and seeded at a density of 3–6 × 10^4^cell cm^−2^. For the first 24 h after replating, culture media was supplemented with 10 µM of Y‐27632 (ROCK inhibitor, TOCRIS). The culture media was changed every day according to manufacturer instructions.

### hiPSC Differentiation into hiPSC‐CM

hiPSC were differentiated toward CM in 6‐well plates when cells reached 80–90% confluency, by temporal modulation of the Wnt/ß‐catenin pathway following a protocol previously described by Maas and colleagues.^[^ ^20^ ^]^ Briefly, hiPSC culture medium was replaced by differentiation medium (RPMI 1640 (RPMI, Gibco, Thermo Fisher Scientific) supplemented with 2% (v/v) B‐27 minus insulin (RPMI/B27‐ins, Gibco, Thermo Fisher Scientific) and CHIR99021 (CHIR, TOCRIS) (Table S6, Supporting Information). After 24 and 48 h, RPMI/B27‐ins was added on top of the medium, and at day 3 the medium was replaced by RPMI/B27‐ins supplemented with 2 µM Wnt‐C59 (TOCRIS). By day 5 and 7 the medium was replaced by RPMI/B27‐ins and RPMI supplemented with 2% (v/v) B‐27 (RPMI/B27, Gibco, Thermo Fisher Scientific), respectively. At day 9, the medium was exchanged by RPMI 1640 Medium with no glucose (Gibco, Thermo Fisher Scientific) supplemented with 2% (v/v) B‐27. At day 11 (when over 80% of the cells were beating), cells were dissociated with TrypLE Select Enzyme (10X), without phenol red (Gibco, Thermo Fisher Scientific) for 15 min at 37 °C. After dissociation, a single cell suspension was prepared in RPMI/B27 supplemented with 10% (v/v) KnockOut Serum Replacement (KOSR, Gibco, Thermo Fisher Scientific) and 10 µM of ROCK inhibitor (hereafter designated as replating medium) and filtered through a 40 µm nylon cell strainer (VWR Cell Strainer, Avantor).

### hiPSC‐CM Expansion in 2D Static Culture System

hiPSC‐CM in replating medium were inoculated in Matrigel‐coated systems (e.g., T‐flasks) (Table S6, Supporting Information), at a density of 2.52 × 10^4^ cell cm^−2^, corresponding to a volumetric concentration of 0.27 × 10^6^ cell mL^−1^ (e.g., 7 mL per 75 cm^2^ T‐flask). After 24 h, the replating medium was replaced by RPMI/B27 supplemented with 2 µM of CHIR, and thereafter changed three times per week. The cells were maintained for up to 11 days at 37 °C in a humidified atmosphere of 5% (v/v) CO_2_ and 95% (v/v) air.

### hiPSC‐CM Expansion as 3D Cell Aggregates in Stirred‐Tank Bioreactors

Process development and optimization of hiPSC‐CM expansion as 3D aggregates was done in small‐scale parallel DasGip cellferm‐pro bioreactor system (Eppendorf AG; working volume: 0.2L), in glass vessel STB equipped with magnetic‐driven trapezoid shaped paddle impellers with long arms as previously described by our group.^[^ ^49^ ^]^ The optimized bioprocess was scaled‐up in 2L SU Univessel (Sartorius AG, working volume: 2L).

### hiPSC‐CM Expansion in 0.2L STB

Before cell inoculation, the DO probe was calibrated with air, saturated. The hiPSC‐CM in replating medium, were inoculated as single cells at a density of 0.27 × 10^6^ cell mL^−1^. Briefly, cells were cultured under defined conditions (Temperature: 37 °C; stirring rate: 80 revolutions per minute (rpm); surface aeration rate: 0.1 volumes per volume of medium (vvm); CO_2_: 5% (v/v) and 95% (v/v) air. Twenty‐four hours after inoculum the culture was supplemented with 2 µM of CHIR and at day 2 (when aggregates were already formed), perfusion of RPMI/B27 supplemented with 2 µM of CHIR was initiated (dilution rate: 0.5 per day) and operated by automated gravimetric control, as described previously by the group,^[^ ^49^ ^]^ until day 11. A sintered glass sparger (of 16–40 µm pore size, Eppendorf) was connected in the outlet perfusion line as cell retention device to prevent aggregate loss. On day 2 onwards, cells were cultured under different conditions, namely aeration via overlay with: i) 95% (v/v) air and 5% (v/v) CO_2_ (i.e., uncontrolled DO; referred to as STB), ii) DO controlled at 47.6% of oxygen concentration in air, saturated (corresponding to 10% oxygen, referred to as STB_10%O_2_), iii) DO controlled at 23.8% of oxygen concentration in air, saturated (corresponding to 5% of oxygen, referred to as STB_5%O_2_), and iv) DO controlled at 71.4% of oxygen concentration in air, saturated (corresponding to 15% of oxygen, referred to as STB_15%O_2_. The DO concentrations were monitored by an optical DO probe and automatically regulated by a cascading DO setpoint at three stepwise levels with Air, N₂, and O₂ using the bioreactor's four‐gas mixing system

For the CHIR, w/o CHIR and STB_Agg condition, hiPSC‐CM suspended in replating medium were aggregated using AggreWell400 6‐well plates (AggreWells, STEMCELL Technologies) at a seeding density of 5 × 10^6^ cell/well. Twenty‐four hours after seeding, the medium was supplemented with 2 µM of CHIR. On day 2, the hiPSC‐CM aggregates were harvested from the AggreWells and transferred into the STB (STB_Agg condition) and in orbitally shaken Erlenmeyer flasks (CHIR and w/o CHIR conditions) agitated at 80 rpm. In STB_Agg, perfusion was initiated immediately after inoculation at a dilution rate of 0.5 per day and cells were cultured for 10 days, and with culture conditions set as previously described for the STB strategy. For CHIR and w/o CHIR conditions, Complete medium exchanges were performed every two days. For the complete medium exchanges, agitation was paused, allowing the cell aggregates to settle by gravity for 10–15 min. After settling, the medium was carefully removed, and fresh medium was then added to the Erlenmeyer to continue hiPSC‐CM expansion.

### hiPSC‐CM Expansion in 2L STB

hiPSC‐CM in replating medium, were inoculated as single cells at a density of 0.27 × 10^6^ cell mL^−1^ in 2 L Univessel SU (Sartorius) STB equipped with two 3‐blade segment impeller (30° angle). Power input per unit volume (P/V) was maintained constant and equal to the 0.2L STB hiPSC‐CM by maintaining the transferred energy from the impeller to the liquid, the stirring rates used in the bioreactor (2L STB) were estimated according to Equation (1) (*Np*: impeller power number; *ρ*: liquid density; *D_i_*: impeller diameter; *N*: agitation speed; and *V*: STB working volume).

(1) $$\begin{array}{ccc} & & P_{0.2L}=P_{2L}\Leftrightarrow {\left(\frac{N^{3}\rho D_{i}^{5}N_{p}}{V}\right)}_{0.2L}={\left(\frac{N^{3}\rho D_{i}^{5}N_{p}}{V}\right)}_{2L}\Leftrightarrow N_{2L}\\ & & ={\sqrt[{3}]{{\left(\frac{N^{3}\rho D_{i}^{5}N_{p}}{V}\right)}_{0.2L}\left(\frac{V}{\rho D_{i}^{5}N_{p}}\right)}}_{2L} \end{array}$$

Briefly, cells were cultured under defined conditions at 37 °C, stirring rate was set to 149 rpm, surface aeration rate at 0.1 volumes per volume of medium (vvm), with 95% (v/v) air and 5% (v/v) CO_2_. Twenty‐four hours after inoculum the culture was supplemented with 2 µM of CHIR and at day 2 (when aggregates were already formed), perfusion of RPMI/B27 supplemented with 2 µM of CHIR was initiated (dilution rate: 0.5 per day) and operated by automated gravimetric control, until day 11. A metal sintered sparger (D12*ID9.1*H90.5 mm, of 30–40 µm pore size, Hengko) was connected in the outlet perfusion line as cell retention device to prevent aggregate loss. From day 2 onwards, cells were cultured under controlled DO at 10% O_2_.

### Cell Concentration and Viability

Viable cell concentration was determined using a NucleoCounter NC‐200 (ChemoMetec), according to the manufacturer's instructions. The “Viability and Cell Count Assay” and the “Viability and Cell Count – A100 and B Assay” protocols, were used for counting of cells cultured in 2D monolayer, and in 3D aggregates, respectively. Expansion factor was calculated based on the ratio of the maximum cell concentration and the hiPSC‐CM concentration at day 0.

For cell viability, cells were stained with 20 µg mL^−1^ of fluorescein diacetate (FDA, Sigma‐Aldrich) and 10 µg mL^−1^ with the DNA‐binding dye propidium iodide (PI, Sigma–Aldrich), followed by observation at the inverted fluorescence microscope (DMI6000, Leica). Cells capable of accumulating the product of FDA metabolization were stained fluorescent green and were alive, while cells‐stained fluorescent red with PI were considered dead.

### Aggregate Size

Cell aggregates sampled from the bioreactor were distributed in 96‐well plates (200 µL/well). Images of cell aggregates were collected (DMI6000 B, Leica) and analyzed in ImageJ open‐source software (Rasband, WS, ImageJ, U. S. National Institutes of Health, Bethesda, MD, USA, http://imagej.nih.gov/ij/, 1997–2012). For estimation of aggregate size, the average Feret diameter of cell aggregates was estimated by analysing several frames collected during live imaging (at least 200 cell aggregates were analyzed per condition).

### Flow Cytometry

Analysis of hiPSC and hiPSC‐CM phenotype was performed by flow cytometry (FC). Briefly, single cell suspensions were prepared after dissociation of cell monolayers/aggregates with Versene (Gibco, Thermo Fisher Scientific) (20 min) or TrypLE Select 10X (15‐45 min) respectively for hiPSC and hiPSC‐CM. A minimum of 0.5 × 10^6^ cells were used per sample. For detection of cell‐surface epitopes (TRA‐1‐60, SSEA‐4 and SSEA‐1), cells were resuspended in the respective primary antibody or isotype control (Table S7, Supporting Information) and incubated at 4 °C for 1 h. For detection of intracellular epitopes (*α*‐actinin and cTnT), cells were fixed and permeabilized using a commercial kit (Inside Stain, Miltenyi Biotec), according to the supplier's instructions, after which they were resuspended in the respective primary antibody or isotype control (Table S7, Supporting Information) and incubated at room temperature (RT, approx. 18–20 °C) for 30 min. When primary unconjugated antibodies were used, an additional incubation with the secondary antibody was performed at RT for 30 min in the dark (Table S7, Supporting Information). For assessment of ROS in the cells, the 2′,7′‐dichlorofluorescin diacetate – Cellular ROS Assay Kit ab113851 (Abcam) was used accordingly to supplier's instructions. Analysis was conducted regarding the relative fold change of the median intensity of ROS present in the sample in terms of its units of fluorescence (u.f). Data was acquired on a BD FACSCelesta Cell Analyzer (BD biosciences) and analyzed in the FlowJo software (FlowJo LLC, http://www.flowjo.com/). A minimum of ten thousand events were registered for each sample on the population of interest.

### Immunofluorescence Microscopy

Cells cultured as 2D monolayer, or 3D aggregates harvested from the bioreactor were fixed in 4% (w/v) paraformaldehyde (PFA) and 4% sucrose in phosphate‐buffered saline (PBS) for 30 min at RT and washed three times with DPBS (+/+). For intracellular marker detection, fixed cells were permeabilized/blocked in DPBS containing 0.1% (v/v) Triton X‐100 and 0.2% (v/v) gelatine from cold water fish skin for 20 min for cells in 2D monolayer or 30 min for 3D aggregates, at RT. Cells were washed three times with DPBS, and incubated with primary antibodies (Table S8, Supporting Information) for 1 h at RT in the dark. For cell aggregates, this was followed by an overnight incubation at 4 °C. Cells were then washed three times with DPBS and incubated in the dark with secondary antibodies (Table S8, Supporting Information) for 1 h at RT, followed by an additional hour in the case of cell aggregates. Two final washing steps with DPBS were performed, after which cell nuclei were counterstained with 4′,6‐diamidino‐2‐phenylindole (DAPI). Finally, samples were mounted with ProLong Glass Antifade Mountant (ProLong, Invitrogen). Immunofluorescence images were obtained with an Inverted Laser Scanning Confocal Microscope (Zeiss LSM 880 with Fast Airyscan, ZEISS), and processed using ImageJ open‐source software (Rasband, WS, ImageJ, U. S. National Institutes of Health, Bethesda, MD, USA, http://imagej.nih.gov/ij/, 1997–2012).

Immunofluorescent images of cell aggregates stained with Ki‐67 were further analyzed, using ImageJ software for quantification of proliferative cells (% of Ki‐67+ cells). Nuclei co‐stained with DAPI and Ki‐67 were counted and then divided by the total number of nuclei within the cell aggregate to calculate the percentage of Ki‐67 positive cells.

### Gene Expression Analysis

Cells were centrifuged at 300 g for 5 min, washed with DPBS, snap‐frozen with liquid nitrogen and kept at −80 °C. mRNA was extracted using the High Pure RNA Isolation kit (Roche) according to manufacturer's instructions. mRNA was quantified using a NanoDrop 2000c spectrophotometer (Thermo Scientific) and cDNA synthesized with 100 ng RNA per sample, using the Transcriptor High Fidelity cDNA Synthesis Kit (Roche). Real time quantitative polymerase chain reaction (RTq‐PCR) was performed using the LightCycler 480 Instrument II 384‐well block (Roche) in the following cycles: incubation at 95 °C for 10 min; 45 cycles of amplification with denaturation at 95 °C for 15 sec, and annealing at 60 °C for 1 min; extension at 72 °C for 5 min. Each sample was run in triplicate and data analyzed in LightCycler 480 Software v1.5.0 (Roche). Gene expression data was normalized to housekeeping genes RPLP0 and GADPH and relative changes were analyzed using the 2^−ΔΔCt^ method.^[^ ^50^ ^]^ Primers used for gene expression analysis are summarized in Table S9 (Supporting Information).

### RNA‐Seq Analysis

Cells were harvested, then stored in TRIzol at −80 °C until further processed. Following RNA extraction, 100 ng of total RNA were used for transcriptomic interrogation using Illumina's Stranded Total RNA Prep Ligation with Ribo‐Zero Plus according to the manufacturer's instructions. Briefly, cytoplasmic and mitochondrial rRNAs as well as beta globin transcripts were depleted from the samples. The remaining RNA was fragmented and reverse‐transcribed. A second strand cDNA synthesis step removed the RNA template while incorporating dUTP in place of dTTP in order to preserve strand specificity. Next, double‐stranded cDNA was A‐tailed, then ligated to Illumina anchors bearing T‐overhangs. PCR‐amplification of the library allowed the barcoding of the samples with 10 bp dual indexes and the completion of Illumina sequences for cluster generation. Libraries were quantified with Qubit dsDNA HS Assay Kit and their profile was examined using Agilent's HS D1000 ScreenTape Assay. Sequencing was carried out in an Illumina NextSeq2000 using paired‐end, dual‐index sequencing (Rd1: 59 cycles; i7: 10 cycles; i5: 10 cycles; Rd2: 59 cycles) at a depth of 50 million reads per sample. Samples were demultiplexed using Illumina bcl2fastq software (v.2.2.0) and aligned to the human genome (GRCh38) with STAR (v2.7.0d) using default parameters. Gene expression quantification was performed using the featureCounts function implemented in the R package Rsubread (v2.4.3) counting uniquely mapped reads with reverse strandness. The Ensembl v103 gene annotation was considered as the reference. The filterbyExpr function implemented in the edgeR package (v3.40.2) was used to filter out genes with low number of counts for downstream analyses. Data normalization, transformation (considering variance stabilizing transformation), principal component analysis and differential expression analyses were performed with the DESeq2 package (v1.38.3) taking the CM differentiation into consideration in the design. To compare STB_10%O2 and STB samples, it considered the Wald test, keeping genes with an FDR of 0.05. Overrepresentation analyses of the differentially expressed genes were performed with enrichR (v3.2) against the “MSigDB_Hallmark_2020”, “KEGG_2021_Human”, “Reactome_2022” and “GO_Biological_Process_2023” databases. To evaluate significant expression changes across maturation (including hiPSC‐CM_d0, hiPSC‐CM_d11 and hiPSC‐CM_Mat), it considered the Likelihood Ratio Test (LRT) in DESeq2 with ≈Differentiation as reduced model. Then, the function degPatterns from the library DEGreport (v1.26.0) was used in regularized log transformed data, obtained with the rlog function, to look for different expression patterns in those genes with an FDR < 0.001. In addition, single sample GSEA (ssGSEA) scores were calculated for the HIF‐1 signaling pathway with the GSVA package (v1.46.0) using the log2‐transformed transcripts per million (logTPMs) as input and “ssgsea” for the method parameter. To compare the scores, it performed a paired t test.

### Transmission Electron Microscopy

Cells cultured as 2D monolayers or 3D aggregates were fixed in 0.1 M phosphate buffer (PB) (pH 7.4) with 2% (v/v) formaldehyde and 2.5% (v/v) glutaraldehyde overnight and washed three times with PB. In the case of cell aggregates, these were embedded in 2% (w/v) low melting point agarose that was solidified and sectioned in smaller pieces. The cells were post‐fixed in PB with 1% (v/v) osmium tetroxide for 30 min on ice under agitation, washed two times with PB, and two times with distilled water (dH_2_O), followed by incubation in water (H_2_O) with 1% (w/v) tannic acid for 20 min on ice. After five washes with dH_2_O, cells were contrasted with H_2_O with 0.5% (w/v) uranyl acetate, at RT for 1 h in the dark and dehydrated in a graded series of ethanol (30%, 50%, 75%, 90%, 100% three times (v/v)). In the case of cell aggregates, infiltrations with 25%, 50%, 75% and 100% Embed‐812 resin in ethanol were made for 1 h 30 min each before leaving them in 100% Embed‐812 resin overnight, at RT. Finally, cells were embedded in Embed‐812 resin, for monolayers, and in Embed‐812 resin in ethanol, for cell aggregates, and sectioned on an ultramicrotome (UC7 Ultramicrotome, Leica) using a diamond knife. Sections were collected on formvar‐coated slot grids, stained with 1% (w/v) uranyl acetate and Reynolds lead citrate for 5 min each, and analyzed on a transmission electron microscope FEI Tecnai G2 Spirit BioTWIN at 120 kV. Images were taken with an Olympus‐SIS Veleta CCD Camera. and were later processed using the ImageJ software.

### Electrophysiological Studies

Intracellular Ca^2+^ propagation transient imaging was employed to measure electrical activity. For hiPSC‐CM_d11 and hiPSC‐CM_Mat, 3D aggregates were loaded with Fura‐2 acetoxymethyl ester (Fura‐2 AM, TEFLabs, Austin, TX, USA) dye for ≈30–40 min prior to mapping, followed by washout. 2D_Mat cultures were loaded with rhod‐2 (TEFLabs, Austin, TX, USA) and Probenecid (TEFLabs, Inc, Austin, TX, USA) at 420 µM for 30 min under incubation conditions. The 3D aggregate cultures were illuminated using two UV LED lights (Nichia, Tokushima, Japan) with a peak output of 400 mW and a peak wavelength of 380 nm. The 2D cultures were illuminated with a filtered green LED light source CBT‐90‐G (peak power output 58 W; peak wavelength 524 nm; Luminus Devices, Billerica, USA). The setup included a plano‐convex lens (LA1951, focal length 25.4 mm; Thorlabs, Newton, NJ, United States), a UV excitation filter (FF01‐380/14; Semrock), and a green excitation filter (D540/25X; Chroma Technology, Bellows Falls, USA). Epifluorescence was captured using an EMCCD camera (Evolve‐128: 128 × 128 imaging pixels, 24 × 24‐µm pixels, 16 bits; Photometrics, Tucson, AZ, USA) equipped with a custom multiband‐emission filter (ET585/50‐800/200 M; Chroma Technology) suitable for Fura‐2AM and rhod‐2 emission in conjunction with a high‐speed camera lens (DO‐2595; Navitar Inc., Rochester, USA) mounted on an acquisition setup with a variable squared field of view from 0.5 mm to 5 mm. Custom MATLAB software, developed based on Micro‐Manager Open‐Source Software, facilitated optical mapping epifluorescence image recording and processing.^[^ ^51^ ^]^ Following a standardized protocol at HGUGM,^[^ ^52^ ^]^ each cardiac spheroid underwent a 5‐second acquisition at increasing frequencies: basal stage and stimulation at 1 Hz, 2 Hz, 3 Hz, and 5 Hz. Subsequently, a final basal acquisition was recorded at a higher macro zoom level. After analysis, flecainide antiarrhythmic drug was administered for 5 min. The complete optical mapping signal post‐processing and electrophysiology related metrics are detailed in the Supporting Information.

### Metabolic Profiling

Cell aggregates were centrifuged at 300 g for 5 min and supernatants were collected and analyzed in Cedex Bio Analyzer (Roche) to calculate the concentration of glucose and lactate. Specific rates of glucose consumption and lactate production were calculated according to the general mass balance equation, Equation (2),

(2) $$q=\frac{\frac{\Delta C}{\Delta t}-D\times \left(C_{in}-C_{out}\right)}{\bar{X_{V}}}$$

(*q*: specific synthesis rate; *ΔC/Δt*: change rate in the supernatant; *D*: dilution rate; *C_in_*: inlet concentration; *C_out_*: outlet concentration; $\bar{X_{V}}$: average of viable cell concentrations during the time period *Δt*).

### Statistical Analysis

Statistical analysis was performed using GraphPad Prism software 9.0.1. Data were shown as mean ± standard deviation (SD) of technical replicates, and as mean ± standard error of mean (SEM) of independent biological replicates. The differences between experimental groups were analyzed by using multiple paired t tests or ordinary one‐way ANOVA followed by post hoc analysis using Tukey's test for multiple comparisons. Moreover, Pearson's correlation coefficient (r) was used to analyze the similarity between the expansion profile in 0.2 L DasGip STB and the 2 L Univessel STB, with the maximum correlation of r = 1 representing the best linear fit between the data. P‐values of *p* ≤ 0.05(*), *p* ≤ 0.01 (**), *p* ≤ 0.001 (***), and *p* ≤ 0.0001 (****), were considered statistically significant.

## Conflict of Interest

The authors declare no conflict of interest.

## Data and Code Availability

Bulk RNA sequencing datasets that were generated for this manuscript are publicly available in NCBI Gene Expression Omnibus (GEO, http://www.ncbi.nlm.nih.gov/geo) under the accession number GSE272037.

## Supporting information



Supporting Information

Supplemental Movie 1

Supplemental Movie 2

Supplemental Movie 3

Supplemental Movie 4

Supplemental Movie 5

Supplemental Movie 6

Supplemental Movie 7

Supplemental Movie 8

## Data Availability

The data that support the findings of this study are available from the corresponding author upon reasonable request.
