# Supplementary material for: Billion‐Scale Expansion of Functional hiPSC‐Derived Cardiomyocytes in Bioreactors Through Oxygen Control and Continuous Wnt Activation
Source: Adv Sci (Weinh). 2025 Jan 23;12(11):2410510. doi: 10.1002/advs.202410510 (PMC11923921; doi:10.1002/advs.202410510)
Supplement: Supplementary file 1 — Supporting Information [file ADVS-12-2410510-s003.docx]

Supporting Information

**Billion‐Scale Expansion of Functional hiPSC‐Derived Cardiomyocytes in Bioreactors Through Oxygen Control and Continuous Wnt Activation**

Pedro Vicente, Lara R. Inocêncio, Asier Ullate-Agote, Ana F. Louro, João Jacinto, Beatriz Gamelas, Olalla Iglesias-García, Patxi San Martin-Uriz, Paula Aguirre-Ruiz, Gonzalo R. Ríos-Muñoz, María Eugenia Fernández-Santos, Alain van Mil, Joost P. G. Sluijter, Felipe Prósper, Manuel M Mazo Vega, Paula M. Alves, Margarida Serra*

**Supplementary Methods**

**Electrophysiological Analysis**

*Mask Contouring:* Before initiating image processing, the segmentation of the image into foreground and background is performed to isolate the region of interest and eliminate background noise. This was achieved manually or using an active contours-based region-growing technique. The "active contour" algorithm efficiently identifies the edges of the sample aggregates (Figure S10E-F), effectively reducing background noise and optimizing the computational time for subsequent processing. Alternatively, the mask can also be manually delineated by the user if necessary.

*Post-processing Filtering:* The acquired fluorescence optical signals often contain noise and artifacts that degrade data quality, complicating analysis and interpretation. Post-processing techniques are employed to enhance the signal-to-noise ratio, eliminate artifacts, and improve the overall signal quality.

*Spatial filtering:* A Gaussian filter with a kernel size of 5×5 is applied to mitigate spatial noise and pixel-to-pixel variations, providing a cleaner and more accurate representation of calcium (Ca²⁺) activity*^[1]^*. This step ensures that the resulting data is more reliable for downstream analyses (see Figure S10B).

*Temporal smoothing:* To reduce high-frequency noise and temporal fluctuations, a Locally Weighted Scatterplot Smoothing (LOWESS) filter is applied. This adaptive smoothing method is robust to outliers and accommodates local variations in the signal. Kernel sizes between 5 and 7 were used in this study (Figure S10C).

*Baseline Correction*: Baseline wandering artifacts were removed using three different methods inspired by O’Shea et al. (2019)*^[2]^*. While 4^th^ and 11^th^-degree polynomial fittings were effective, the “tophat” method showed superior performance. In this approach, a sliding window is used to estimate the baseline signal, which is then subtracted from the observed data. This correction preserves the amplitude and shape of calcium transients while normalizing the baseline (Figure S10D).

*Signal Normalization and Temporal/Frequency Analysis:*

*Normalization (ΔF/F₀):* Signals were normalized using the equation:

$$\frac{\Delta F}{F_{0}}=\frac{F_{t}-F_{0}}{F_{0}} ,$$

where $F_{t}$​ represents the fluorescence intensity at time $t$, and $F_{0}$​ is the average fluorescence calculated over a baseline period. The baseline period was defined as the 0.2-second window preceding activations, during which the baseline activity was averaged^[3]^. Normalized signals ($\Delta F$/$F_{0}$​) are expressed as a percentage, enhancing the comparability of calcium transients across regions and experiments.

*Inter-Beat Interval:* The inter-beat interval (IBI) was calculated as the average time interval between consecutive calcium transient (CaT) activations. To determine the timing of each activation, the local activation time (LAT) was identified for each transient using the method described by Ríos-Muñoz et al. (2018)^[4]^. Specifically, the LAT was defined as the time point corresponding to the maximum first derivative of the fluorescence signal (dF/dt), representing the steepest rise in fluorescence intensity during activation.

The calculated IBIs were then analyzed to assess rhythm regularity and temporal characteristics across experimental conditions.

*Dominant frequency:* To analyze the dominant frequency (DF), the "pwelch" function was used to estimate the power spectral density (PSD) of the fluorescence signals. A Hamming window with a length of 2⋅fs was applied, computing the PSD over a frequency range of $f_{s}$ to $2\cdot f_{s}$ ​ with a resolution of $f_{s}$​. The detectable DF range was set to [0.4, 15 Hz], with the DF defined as the frequency peak with the highest power within this range. A minimum power threshold of 0.01 was applied; pixels with PSD values below this threshold were excluded from the analysis as they lacked significant signal power (Figure S11). To create the global DF map, the analysis was performed for each valid pixel in the signal. The resulting DF values were visualized using a color-coded map, where colors corresponded to the dominant frequency values across different regions of the aggregate. Additionally, a histogram of the DF values was generated to illustrate their distribution, including the total DF average and standard deviation. This combination of the DF map and histogram facilitates visualization and comparison of DF distribution across the aggregate, offering valuable insights into spatial heterogeneity (Figure S12).

*Calcium Transient (CaT) Analysis*: The analysis of calcium transients (CaTs) involves defining key parameters to characterize their dynamics. One of the primary parameters is the Rise Time (RT), which was measured as the time required for the calcium upstroke to rise from 10% to 90% of its maximum value from ΔF/F_0_. Additionally, the durations of the calcium transient from t_0 (initiation of the transient) to 20%, 50%, 70%, and 90% of repolarization (indicative of calcium extrusion) are calculated and denoted as CaTD20, CaTD50, CaTD70, and CaTD90, respectively.

*Calcium transient alignment:* For analysis, calcium transients were aligned to a common reference point. This alignment process uses Local Activation Times (LATs) derived from the robust method described by Ríos-Muñoz et al. (2018)^[4]^. The MATLAB function ‘alignsignals’ is then employed to iteratively minimize the temporal discrepancies between transients, ensuring accurate alignment. Median values of the aligned signals are computed instead of averages, as medians are more robust against outliers. A complete aligned and processed calcium transient analysis is illustrated in Figure S13, showing the calculated median, standard deviation, and key reference points.

*Calcium transient reference points:* Key reference points of the calcium transient were identified using the LAT and peak positions:

- - Upstroke start point: As mentioned above, determined as the maximum and minimum of the first derivative.
  - Using the LAT and the peak as fiducial positions, the points at 10% and 90% of the calcium upstroke and 20%, 50%, 70%, and 90% of calcium extrusion were computed. From these reference points, the Rise Time (RT) and all CaTD parameters are derived accordingly (Figure S13B).

*Global CaTD maps:* To analyze the spatial distribution of CaTD values, the process described above is applied to every valid pixel within the acquisition mask. The mask is created at the start of the analysis to optimize computational efficiency and exclude non-relevant pixels. For each pixel, the parameters CaTD20, CaTD50, CaTD70, and CaTD90 are computed and visualized as global maps, providing a clear representation of spatial variations in calcium dynamics.

These graphical representations allow for a comparative understanding of calcium transient durations across different regions of the cell population or experimental conditions. Additionally, mean values of all calculated parameters are determined and subsequently used in statistical analyses to draw conclusions and compare experimental groups (Figure S14). Other parameters like the DF, F₀, rise time, max ΔF/F₀, and IBI maps are also displayed.

**Supplementary Figures**


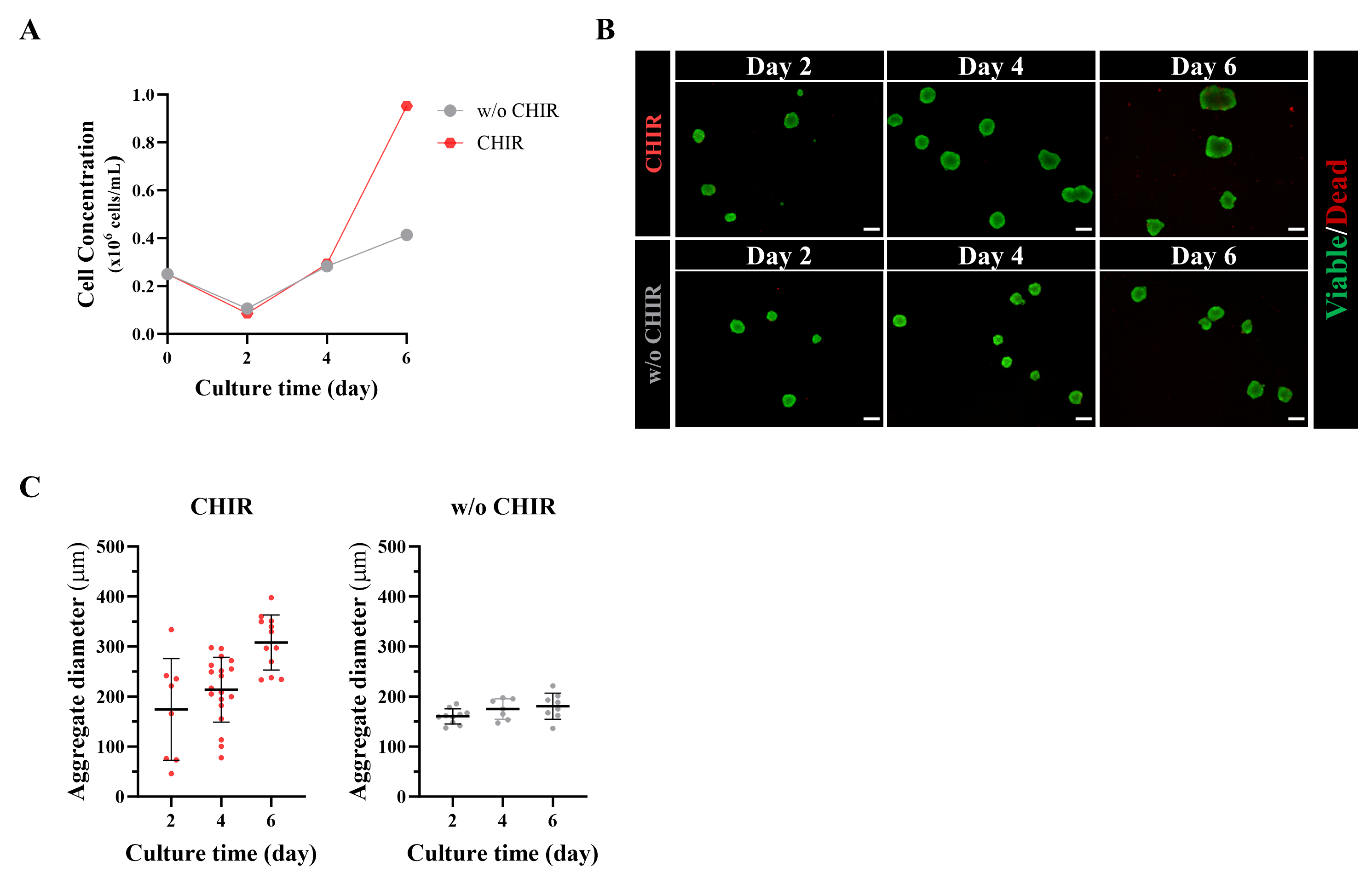


**Figure S1.** Wnt pathway activation promotes hiPSC-CM aggregates expansion. (A) Viable cell growth profile throughout hiPSC-CM expansion, for CHIR (red) and w/o CHIR (grey) conditions. (B) Fluorescence images of hiPSC-CM aggregates in the suspension culture conditions with CHIR and w/o CHIR at days, 2, 4 and 6, stained with FDA (live cells, green) and PI (dead cells, red). Scale bar = 200 µm. (C) Average hiPSC-CM aggregate diameter estimated at days 2, 4, 6 in CHIR and w/o CHIR conditions.


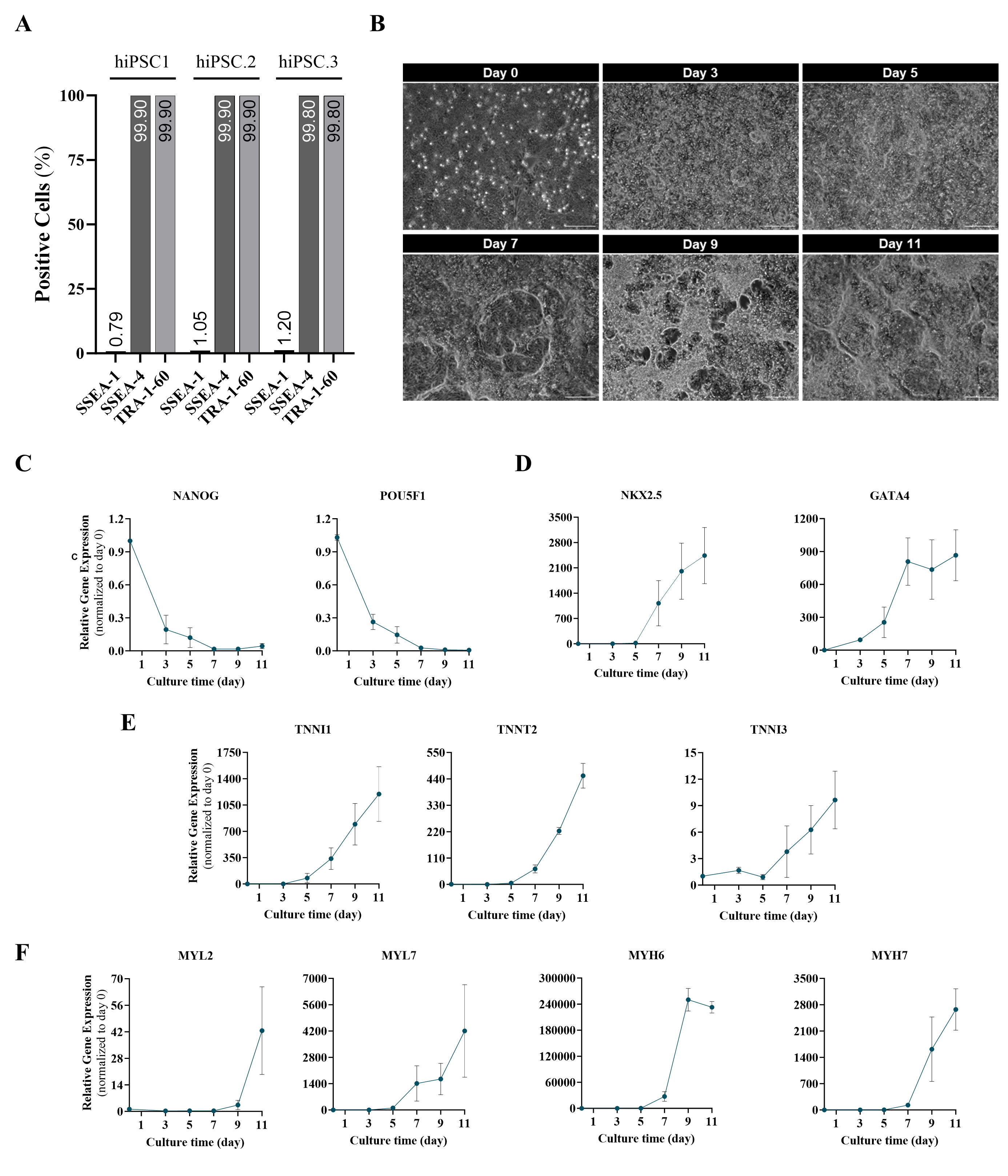


**Figure S2.** Differentiation of hiPSC into CM. (A) Flow cytometry analysis of pluripotency markers SSEA-4 and TRA-1-60, and of mesoderm marker SSEA-1, performed at day 0 of the differentiation protocol for hiPSC.1, hiPSC.2 and hiPSC.3 (B) Representative phase contrast images of hiPSC.1 (day 0) morphology and of the subsequential stages of the cardiac differentiation process at days 3, 5, 7, 9, and 11. Scale bars = 200 µm. (C-F). Relative gene expression of (C) pluripotency genes, NANOG and POU5F1, (D) cardiac progenitor genes GATA4 and NKX2-5, and cardiomyocyte related genes (E) TNNI1, TNNT2, TNNI3, (F) MYL2, MYL7, MYH6, and MYH7 throughout the differentiation process (n=3). Relative gene expression was determined using the 2^-ΔΔCT^ method relatively to day 0 of the differentiation, normalized to the housekeeping genes RPLP0 and GAPDH.


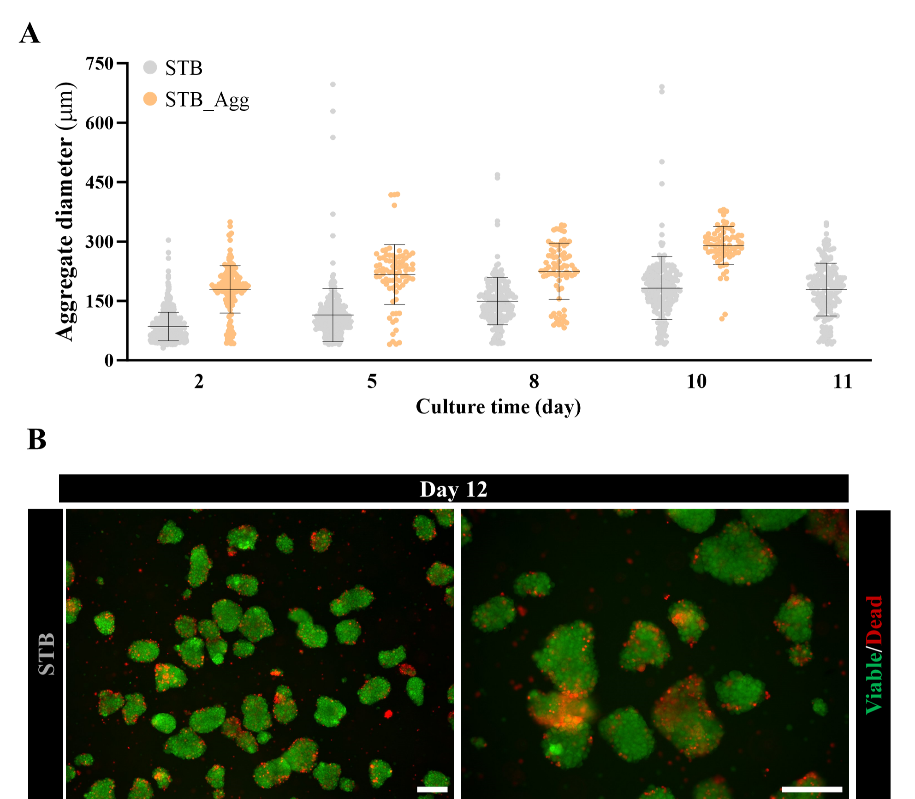


**Figure S3.** hiPSC-CM aggregate size and viability in STB. (A) Average hiPSC-CM aggregate diameter estimated at days 2, 5, 8, 10, 11 in STB and at days 2, 5, 8, 10 in STB_Agg (more than 200 aggregates per condition were analysed). (B) Fluorescence images of cell aggregates in STB at day 12, stained with fluorescein diacetate (FDA, live cells, green) and Propidium iodide (PI, dead cells, red). Scale bar = 200 µm. n_STB_=3, n_STB_Agg_=1.


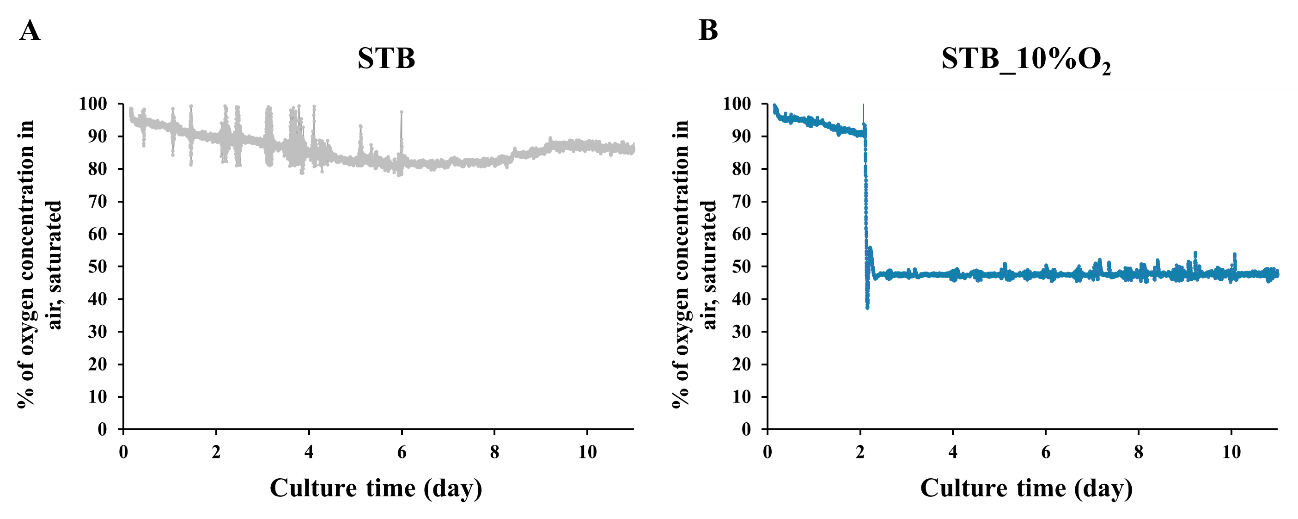


**Figure S4.** Online monitoring trend lines of DO (% air saturation) for STB (A) and STB_10%O_2_ (B) conditions.


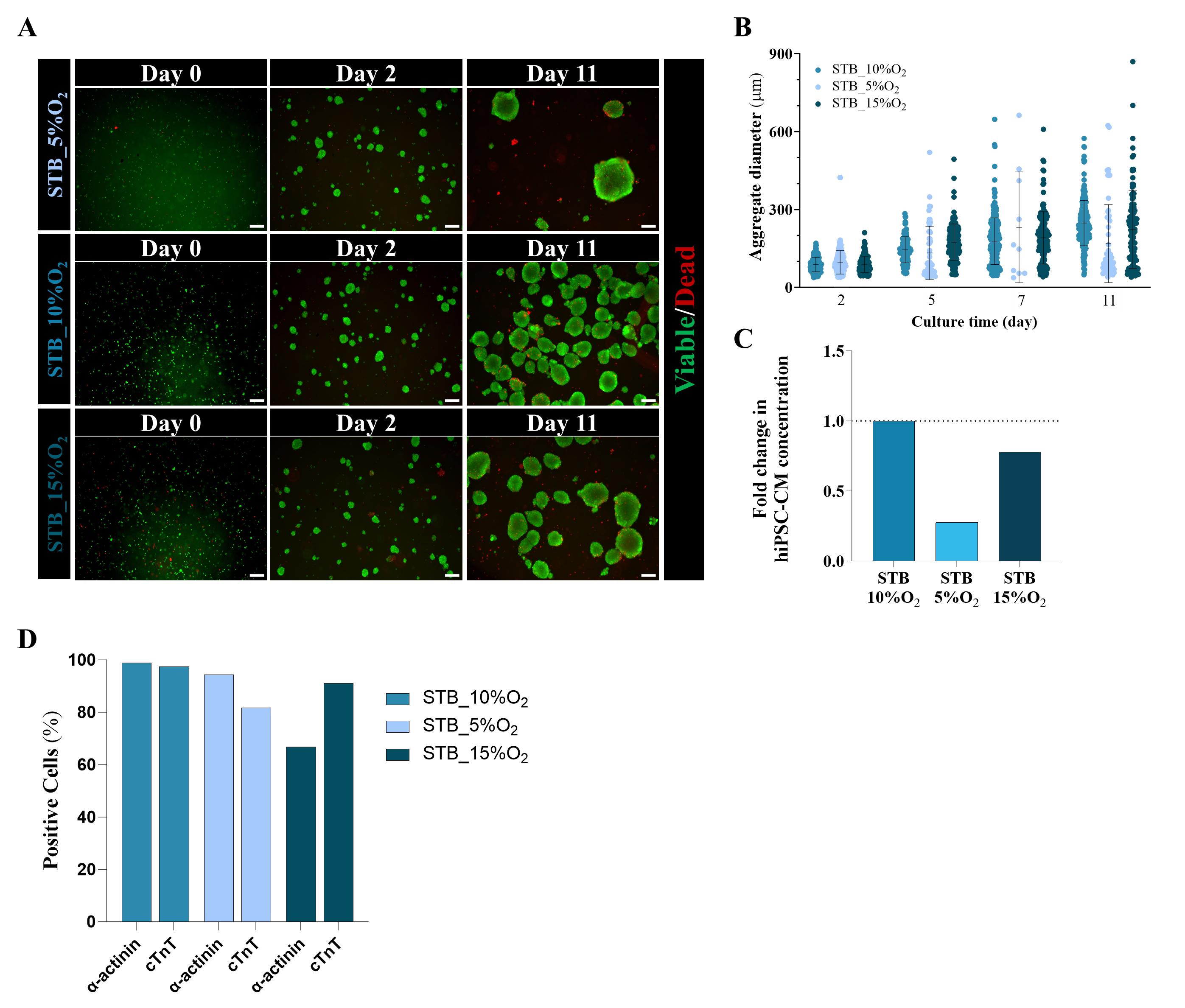


**Figure S5.** Mild hypoxia (10% O_2_) supports higher hiPSC-CM aggregate viability and proliferation in stirred-tank bioreactors. (A) Fluorescence images of cell aggregates in the suspension culture conditions for STB_5%O_2_, STB_10%O_2_ and STB_15%O_2_ at days 0, 2, 11 stained with fluorescein diacetate (FDA, live cells, green) and Propidium iodide (PI, dead cells, red). Scale bar = 200 µm. (B) Average hiPSC-CM aggregate diameter estimated at days 2, 5, 7 and 11 in STB_5%O_2_, STB_10%O_2_ and STB_15%O_2_. (C) Fold change in hiPSC-CM concentration, at end of expansion (day 11), compared to the STB_10%O_2_ condition. (D) Flow cytometry analysis of cardiomyocyte-specific markers α-actinin and cTnT, performed at the last day of hiPSC-CM expansion (day 11).


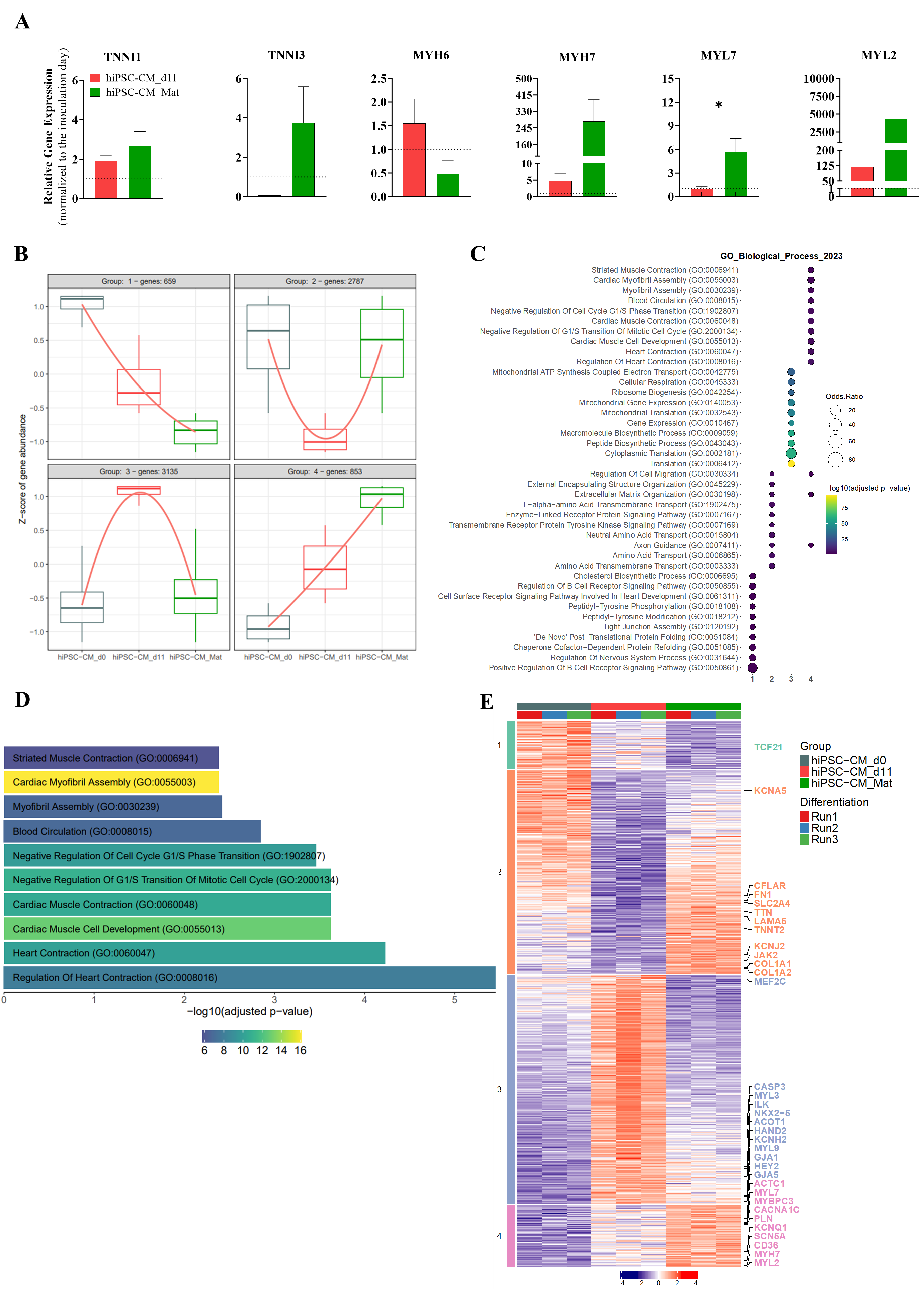


**Figure S6.** Transcriptome analysis reflects the cellular changes that occur during hiPSC-CM differentiation, expansion and maturation. (A) Relative gene expression of CM-related genes compared to STB inoculum. (B) Representation of expression patterns for differentially expressed genes (adjusted p-value < 0.001) from a Likelihood ratio test (LRT). (C) Dotplot representing the top 10 significant GO Biological Processes in an overrepresentation analysis for each gene expression pattern. Size represents the Odds Ratio and color codes the -log10(adjusted p-value). (D) Bar plot representing the top 10 significant GO Biological processes in Group 4 upregulated in a overrepresentation analysis in hiPSC-CM_Mat compared to hiPSC-CM_d11. (E) Heatmap representing z-score scaled gene expression values for differentially expressed genes from the LRT. The heatmap rows are color coded according to the gene expression pattern, labelling certain cardiac related markers of interest colored by pattern. Error bars represent SEM; *, p < 0.05; by paired t test.


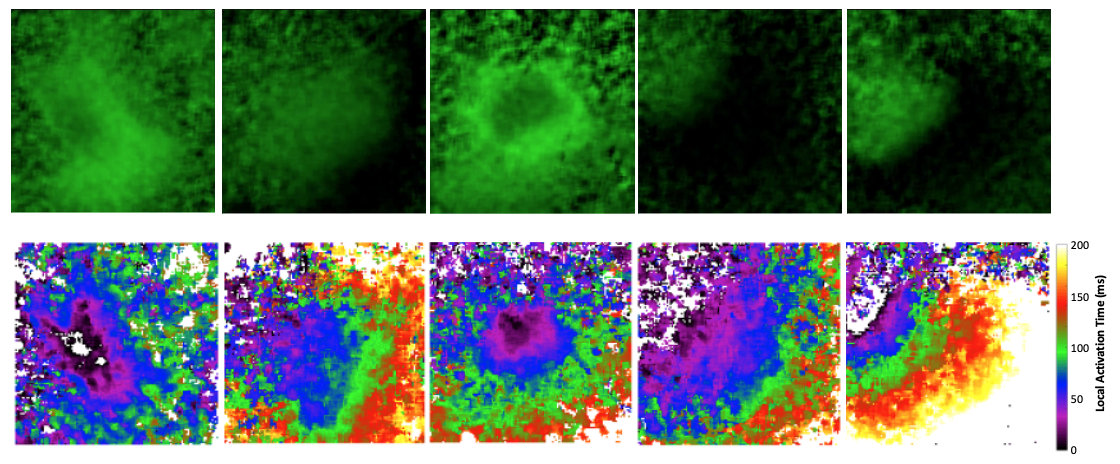


**Figure S7.** Processed fluorescence optical mapping recordings and corresponding isochronal maps for 2D mature cardiomyocyte experiments. **Top Row:** Single frame from the processed fluorescence recordings, showing calcium transient propagation in all experiments across the field of view (FOV: 17.5 mm × 17.5 mm). **Bottom Row:** Isochronal maps depicting the propagation of the activation pattern, with time represented in milliseconds (ms). These maps highlight spatial and temporal patterns of calcium wave propagation.


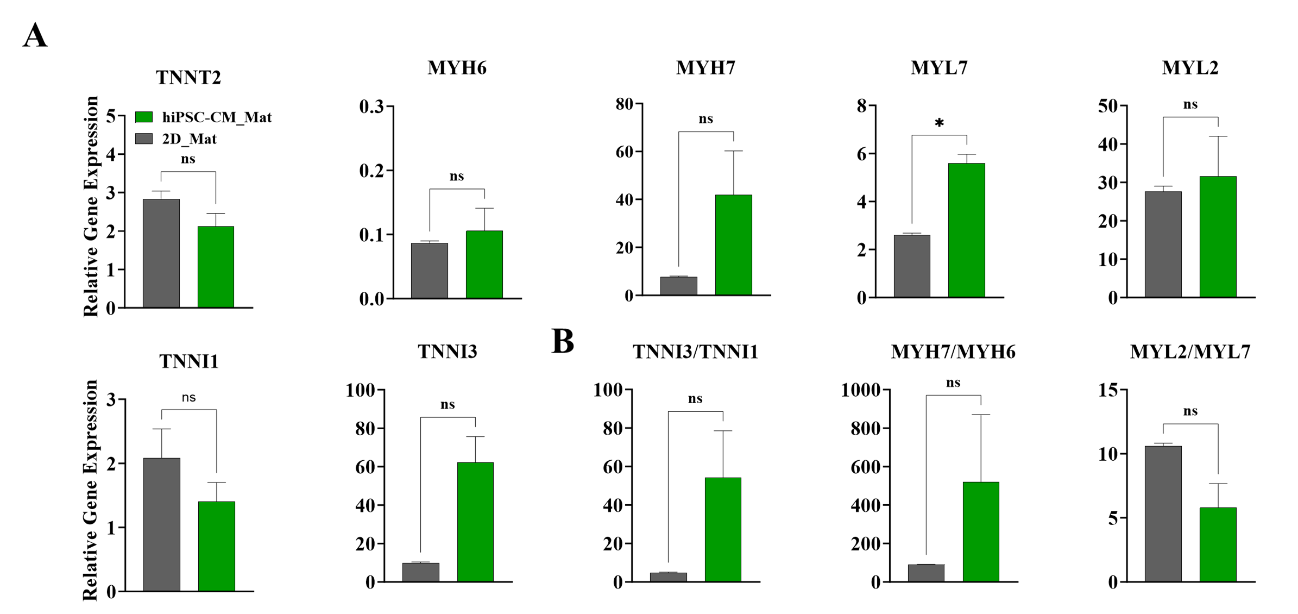


**Figure S8.** hiPSC-CM expanded in both 2D static and STB systems mature in culture after two weeks maturation (A) Relative gene expression of CM-related genes normalized to hiPSC-CM after 11 days of expansion. (B) Ratios of TNNI3 to TNNI1, of MYH7 to MYH6, and of MYL2 to MYL7 gene expression compared to hiPSC-CM after 11 days of expansion.


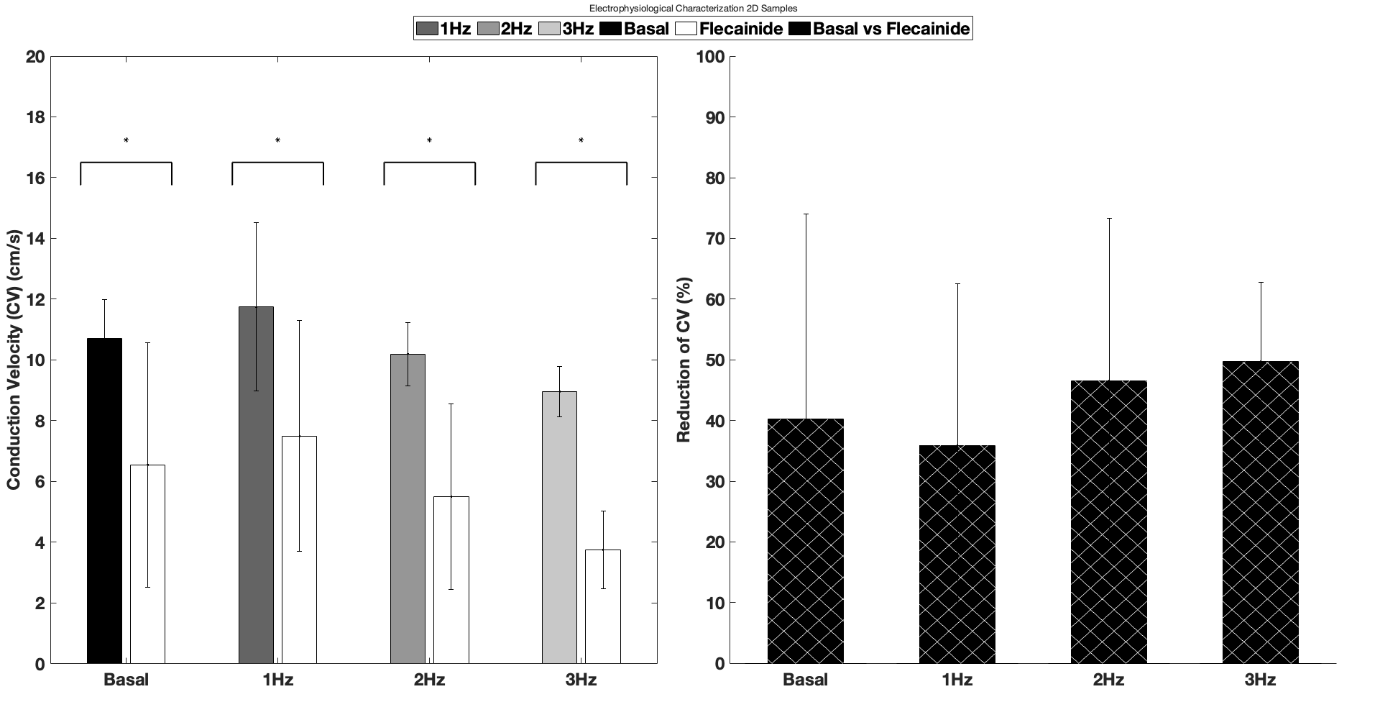


**Figure S9.** Conduction velocity (CV) measurements before and after flecainide treatment under basal conditions and during external pacing at 1Hz, 2Hz, and 3Hz. **(Left Panel):** CV values under different conditions, with significant differences between basal and paced conditions indicated by asterisks (*). **(Right Panel):** Percentage reduction in CV after flecainide treatment across all conditions, showing consistent drug-induced reduction. Data are presented as mean ± standard deviation. Statistical significance was determined using paired t-tests, with p-values < 0.05 marked in the left panel.

| 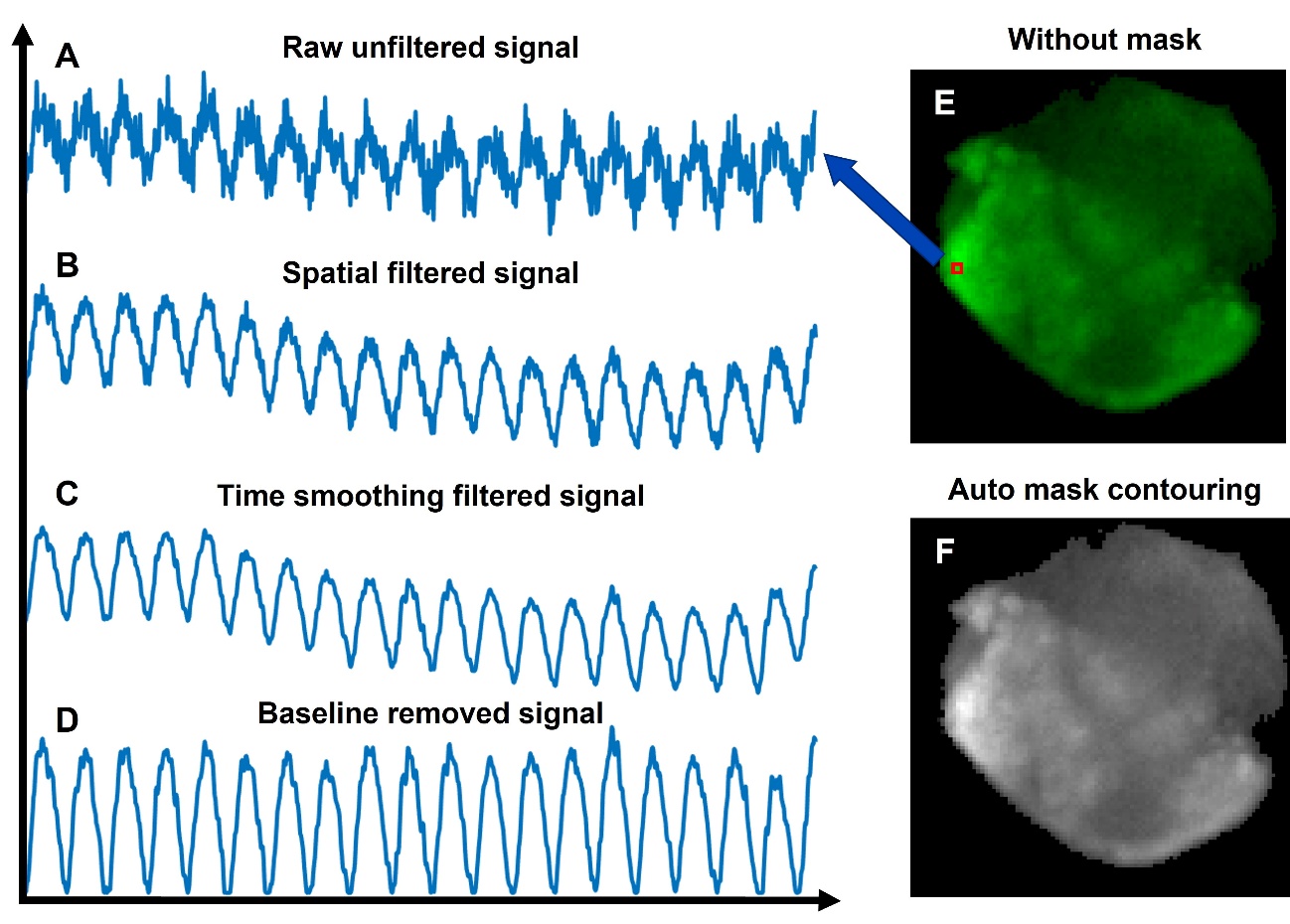 |
| --- |
| **Figure S10.** Signal Post-Processing Summary. (A) Unfiltered signal from the highlighted red pixel shown in (E). (B) Signal after spatial filtering. (C) Signal after temporal smoothing. (D) Baseline-corrected signal using the ‘tophat’ method. (E) Visualization of the unfiltered acquisition without an applied mask, with the red pixel indicating the location of signals in (A-D). (F) Unfiltered acquisition visualization with an auto-computed mask using the "active contour" algorithm. Fluorescence signals are in arbitrary units, and the temporal axis represents 10 seconds of acquisition. |

| **A** |
| --- |
| **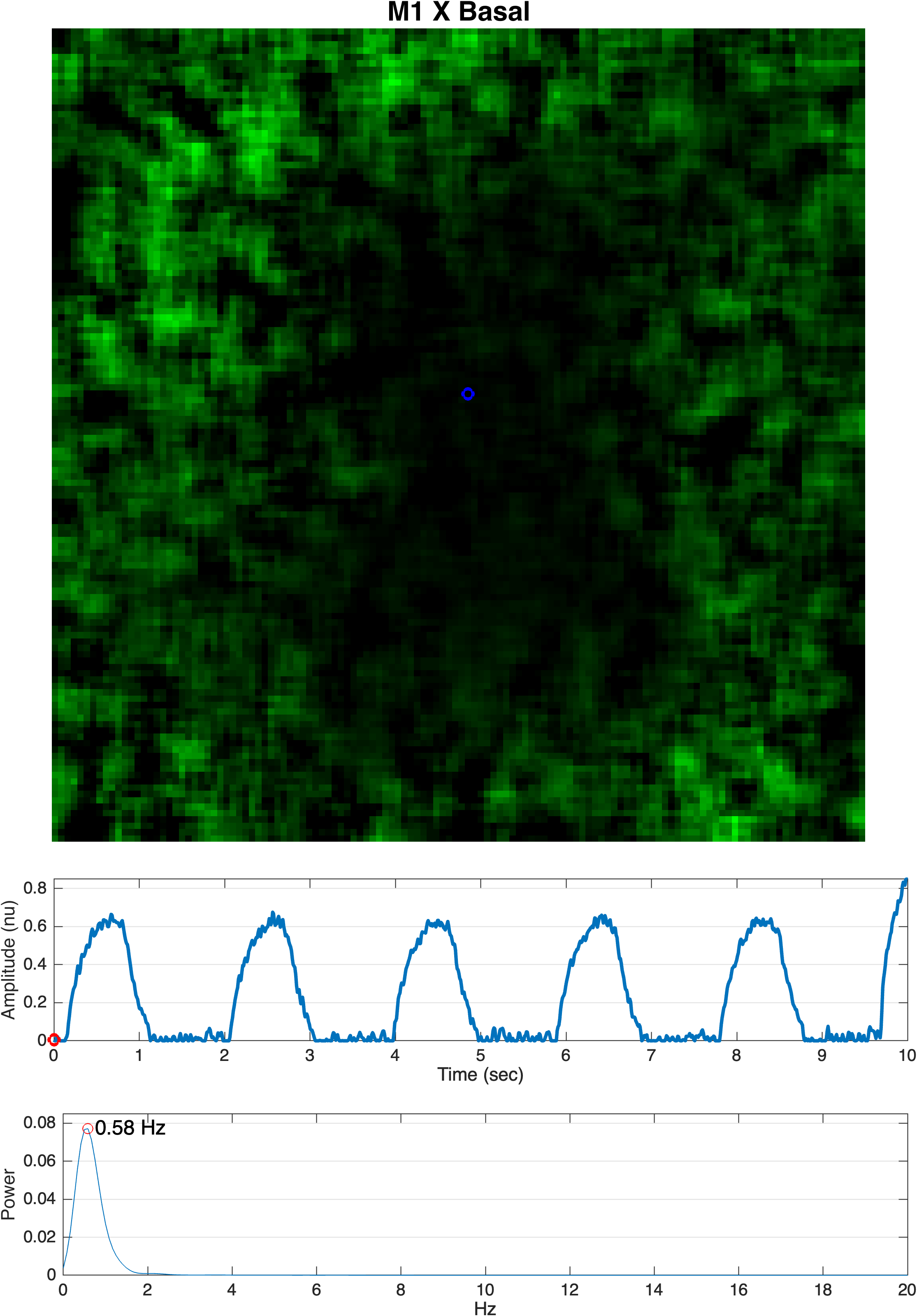** |
| **B** |
| **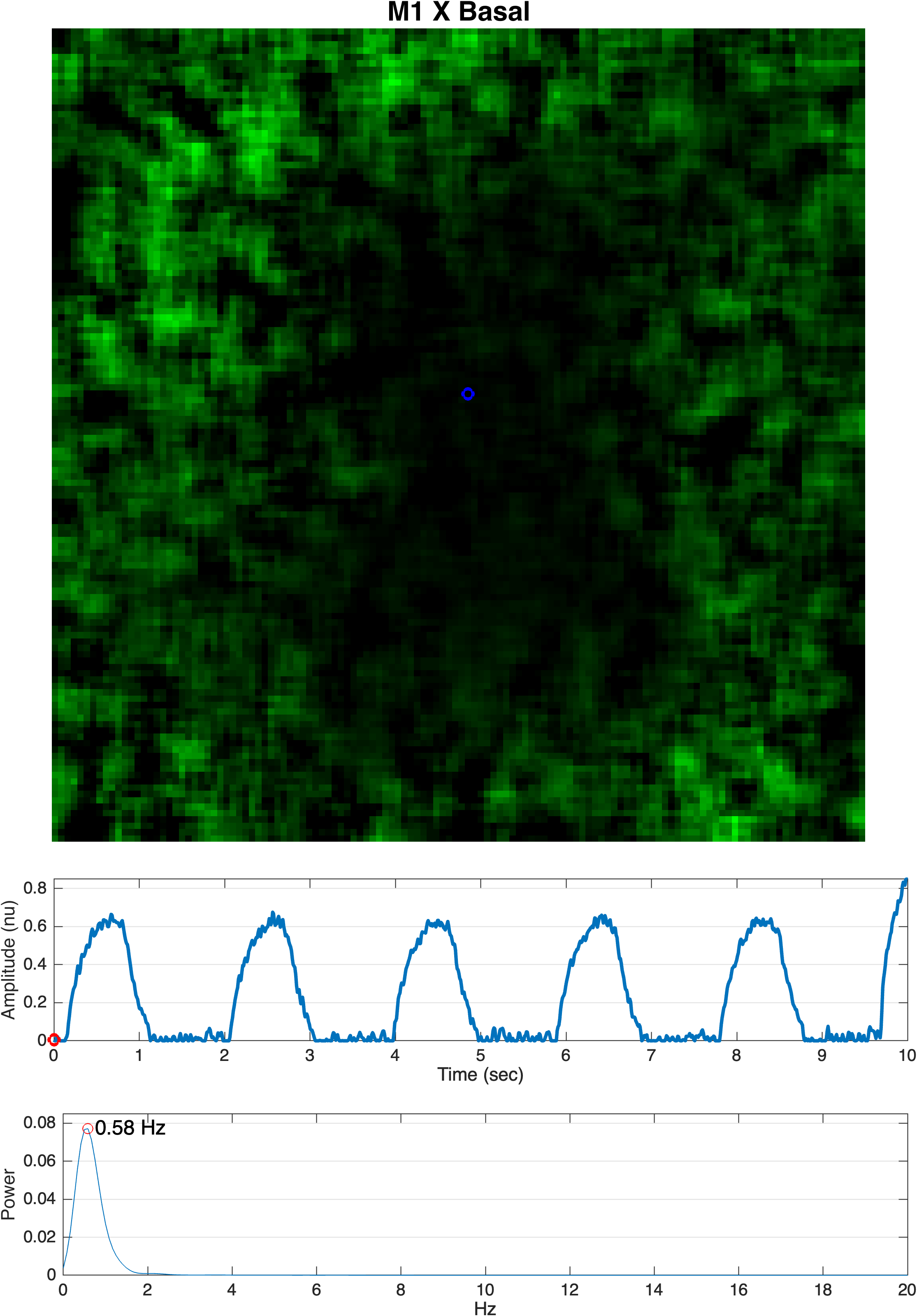** |
|  |
| **Figure S11.** Dominant Frequency calculation. (A) Filtered fluorescence signal. (B) Frequency spectrum density of a signal with DF of 0.58 Hz. |

| **A** | **B** |
| --- | --- |
| 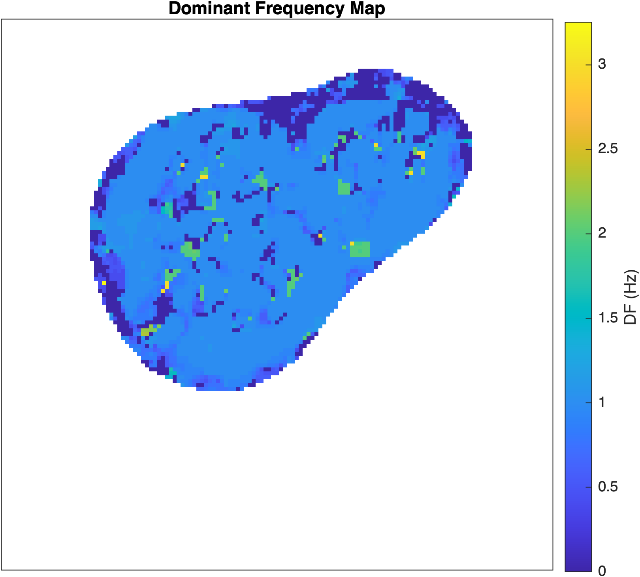 | 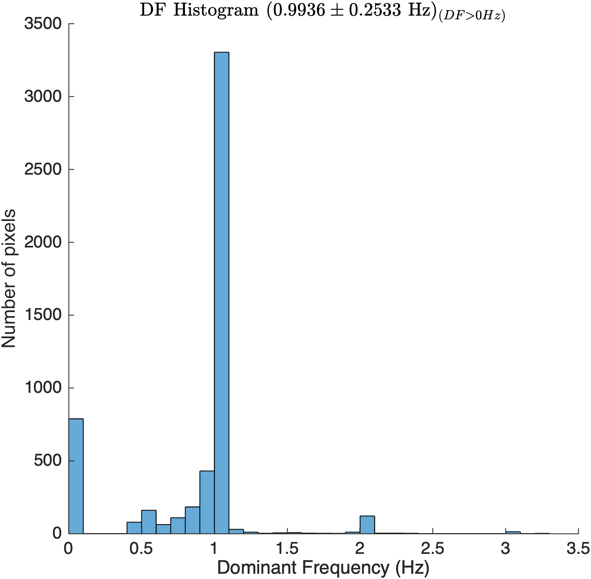 |

**Figure S12.** Global Dominant Frequency Representations. (A) DF map with color-coded values for different regions of the aggregates. (B) Histogram showing the DF distribution across all pixels, including the total DF average and standard deviation.

| **A** | **B** |
| --- | --- |
| *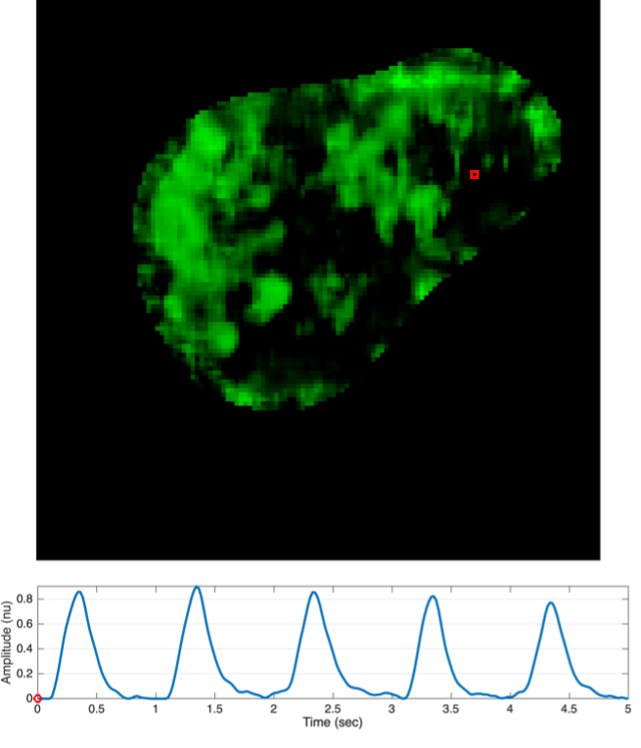* | *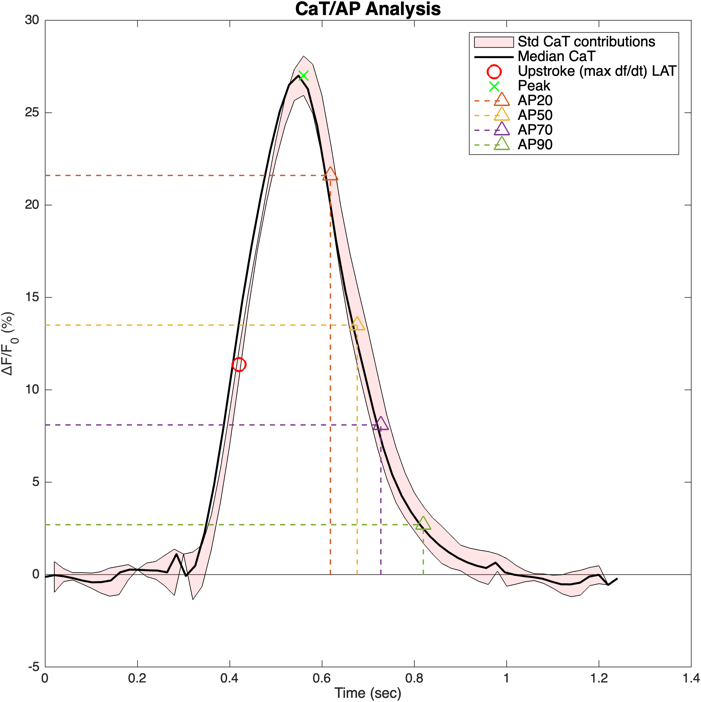* |

**Figure S13.** Calcium Transient Alignment. (A) Post-processed frame and filtered signal for the selected pixel in red. (B) Aligned calcium transients along with calculated median and average values displaying CaT key parameters.

| **A** | **B** |
| --- | --- |
| 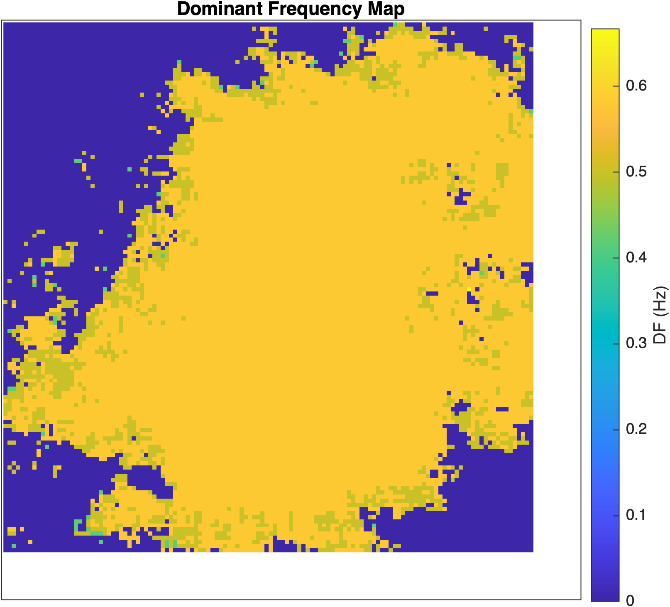 | 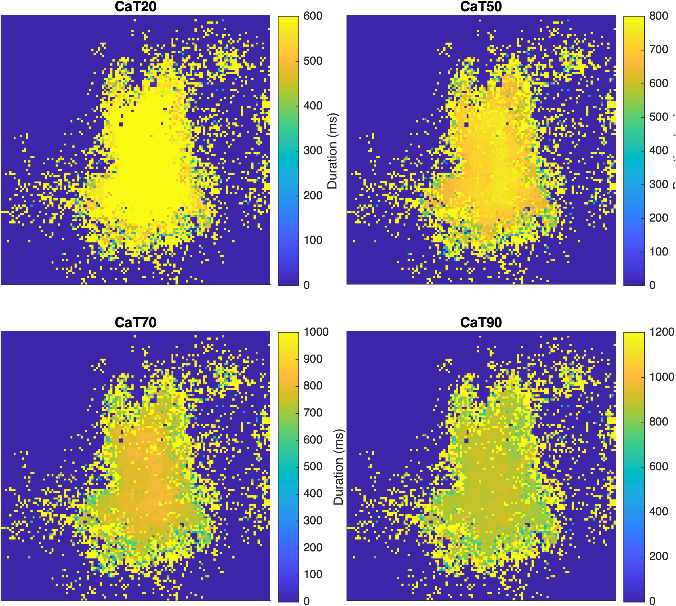 |
|  |  |
| **C** | **D** |
| 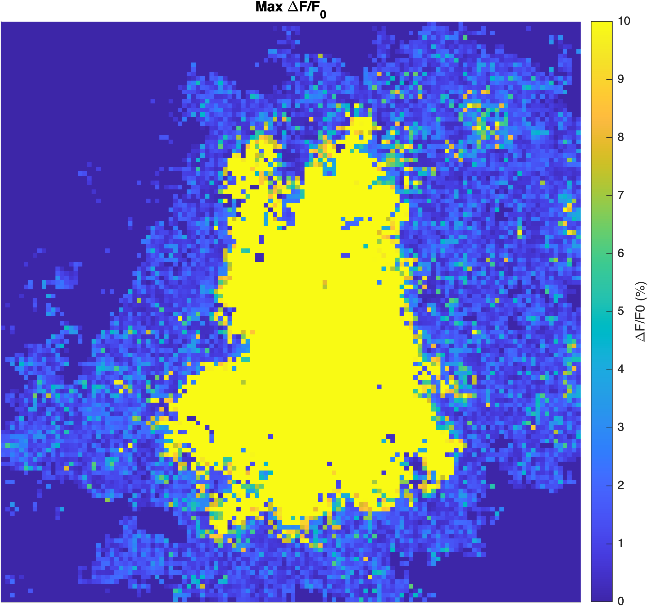 | 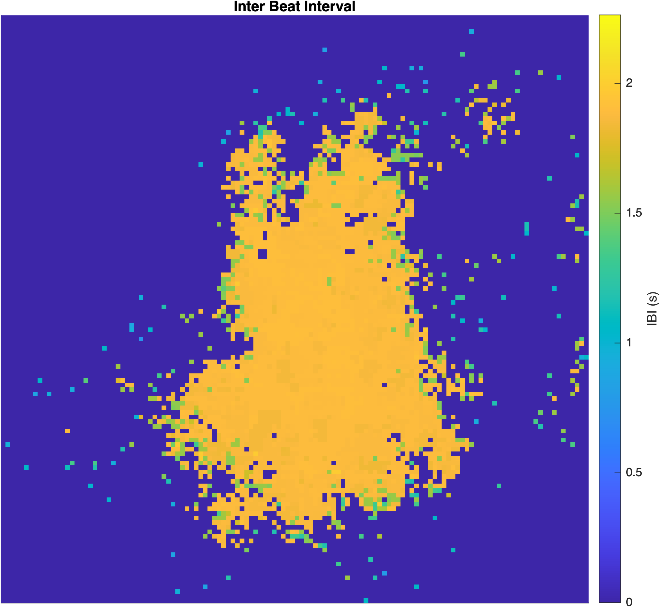 |
|  |  |
| **Figure S14.** Global Calcium Transient Maps. (A). Dominant frequency map. (B) Calcium transient duration (CaTD) maps for CaTD20, CaTD50, CaTD70 and CaTD90 respectively. (C) Max ΔF/F₀ per pixel map. (D) Inter Beat Interval (IBI) in seconds. CaTD and IBI pixels with no DF activity or Max ΔF/F₀<3 are nor displayed to improve the quality of the measurements. | |

**Supplementary Tables**

**Table S1.** Metrics of hiPSC-CM expansion process in 2D_static and as 3D aggregates in STB at different culture conditions. Estimated number of T-flasks (75cm^2^) and 2L STB vessels required for the production of 4 × 10^9^ cells) in each strategy are also presented.

|  | **2D_Static** | **STB** | **STB_10%O_2_** |
| --- | --- | --- | --- |
| Maximum total cell concentration (×10^6^ cell/mL) | 0.8 ± 0.1 | 1.1 ± 0.4 | 2.1 ± 0.1 |
| hiPSC-CM Expansion Factor | 3.9 ± 1.1 | 4.1 ± 1.2 | 9.2 ± 1.4 |
| **Production of 4 × 10^9^ hiPSC-CM (**number of T-flask (75cm^2^) or STB (2L) required) | 715 | 2 | 1 |

**Table S2.** 2D matured conduction velocity (CV) measurements under basal conditions and during external pacing at 1Hz, 2Hz, and 3Hz, before and after treatment with the antiarrhythmic drug flecainide. CV was significantly reduced after flecainide treatment across all conditions, with percentage reductions and associated p-values indicating statistical significance. Data are presented as mean ± standard deviation. Statistical significance was determined using paired t-tests, with p-values < 0.05 highlighted in bold.

|  | **CV (cm/s)** | **CV after Flecainide (cm/s)** | **CV Reduction (%)** | **P-value** |
| --- | --- | --- | --- | --- |
| Basal | 10.7±1.3 | 6.5±4.0 | 40.2±33.8 | **0.0295** |
| 1Hz Stimulation | 11.7±2.8 | 7.5±3.8 | 35.9±26.6 | **0.0392** |
| 2Hz Stimulation | 10.2±1.0 | 5.5±3.0 | 46.5±26.8 | **0.0137** |
| 3Hz Stimulation | 9.0±0.8 | 3.7±1.3 | 49.7±13.1 | **0.0036** |

| **Condition** | **Rhythm** | **DF (Hz)** | **CaTD20 (ms)** | **CaTD50 (ms)** | **CaTD70 (ms)** | **CaTD90 (ms)** | **RiseTime (s)** | **F0 (a.u.)** | **Max ΔF/F0 (%)** | **IBI (s)** | **CV (mm/s)** |
| --- | --- | --- | --- | --- | --- | --- | --- | --- | --- | --- | --- |
| **2D_Mat** | Basal | 0.86 ± 0.20 | 542.38 ± 145.70 | 634.52 ± 147.63 | 720.79 ± 154.61 | 837.50 ± 169.25 | 0.25 ± 0.03 | 0.18 ± 0.02 | 6.47 ± 5.50 | 1.22 ± 0.31 | 106.94 ± 11.47 |
| **2D_Mat** | 1Hz | 1.00 ± 0.00 | 473.37 ± 40.71 | 558.95 ± 35.44 | 632.65 ± 26.90 | 729.95 ± 11.75 | 0.27 ± 0.03 | 0.20 ± 0.01 | 2.88 ± 0.38 | 1.00 ± 0.00 | 117.37 ± 24.80 |
| **2D_Mat** | 2Hz | 1.39 ± 0.48 | 383.95 ± 112.38 | 463.90 ± 117.92 | 527.16 ± 128.89 | 610.98 ± 143.42 | 0.21 ± 0.03 | 0.20 ± 0.02 | 2.86 ± 0.43 | 1.00 ± 0.00 | 101.91 ± 9.29 |
| **2D_Mat** | 3Hz | 1.25 ± 0.25 | 440.12 ± 96.54 | 511.91 ± 102.85 | 564.53 ± 109.48 | 628.96 ± 117.00 | 0.24 ± 0.00 | 0.20 ± 0.03 | 2.07 ± 0.16 | 0.83 ± 0.15 | 109.29 ± 25.63 |
| **2D_Mat** | Flecainide | 0.65 ± 0.18 | 827.38 ± 229.63 | 970.68 ± 245.53 | 1082.33 ± 255.12 | 1206.00 ± 263.32 | 0.32 ± 0.03 | 0.18 ± 0.04 | 4.04 ± 3.14 | 1.54 ± 0.33 | 65.43 ± 35.96 |
| **hiPSC-CM_d11** | Basal | 1.03 ± 0.03 | 305.15 ± 33.63 | 373.12 ± 33.46 | 434.25 ± 32.81 | 541.16 ± 25.61 | 0.31 ± 0.04 | 0.17 ± 0.02 | 6.02 ± 1.46 | 1.07 ± 0.01 | - |
| **hiPSC-CM_d11** | 1Hz | 0.86 ± 0.07 | 349.18 ± 8.53 | 417.90 ± 7.73 | 476.62 ± 4.39 | 599.68 ± 0.56 | 0.39 ± 0.01 | 0.16 ± 0.01 | 7.97 ± 2.58 | 1.34 ± 0.16 | - |
| **hiPSC-CM_d11** | 2Hz | 0.85 ± 0.17 | 405.99 ± 119.44 | 475.99 ± 128.03 | 539.24 ± 130.73 | 669.84 ± 127.52 | 0.34 ± 0.02 | 0.19 ± 0.00 | 4.74 ± 0.00 | 1.05 ± 0.00 | - |
| **hiPSC-CM_d11** | 4Hz | 0.94 ± 0.08 | 290.22 ± 14.79 | 360.68 ± 18.64 | 436.11 ± 19.23 | 593.49 ± 7.91 | 0.31 ± 0.02 | 0.20 ± 0.01 | 5.93 ± 0.84 | 1.17 ± 0.07 | - |
| **hiPSC-CM_d11** | Flecainide | - | - | - | - | - | - | - | - | - | - |
| **hiPSC-CM_Mat** | Basal | 0.73 ± 0.13 | 595.21 ± 87.20 | 733.73 ± 84.32 | 833.03 ± 85.80 | 999.92 ± 107.62 | 0.28±0.06 | 0.18 ± 0.06 | 4.30 ± 1.87 | 1.50 ± 0.16 | - |
| **hiPSC-CM_Mat** | 1Hz | 0.94 ± 0.14 | 493.48 ± 73.07 | 617.12 ± 78.40 | 687.60 ± 91.03 | 788.09 ± 126.20 | 0.27±0.04 | 0.19 ± 0.03 | 3.22 ± 0.47 | 1.17 ± 0.23 | - |
| **hiPSC-CM_Mat** | 2Hz | 0.85 ± 0.18 | 553.30 ± 132.46 | 687.53 ± 137.63 | 766.36 ± 154.95 | 882.60 ± 198.06 | 0.27±0.04 | 0.19 ± 0.03 | 3.76 ± 1.40 | 1.28 ± 0.28 | - |
| **hiPSC-CM_Mat** | 3Hz | 1.03 ± 0.20 | 490.03 ± 117.80 | 607.23 ± 126.91 | 671.53 ± 137.70 | 751.92 ± 159.67 | 0.25±0.05 | 0.20 ± 0.02 | 3.05 ± 0.42 | 1.08 ± 0.22 | - |
| **hiPSC-CM_Mat** | Flecainide | 0.59 ± 0.11 | 801.61 ± 171.47 | 1068.86 ± 180.16 | 1232.03 ± 208.24 | 1365.87 ± 214.26 | 0.32 ± 0.07 | 0.21 ± 0.05 | 3.88 ± 2.27 | 1.75 ± 0.16 | - |

**Table S3.** Summary of electrophysiological and calcium transient parameters for 2_Mat, hiPSC-CM_d11 and hiPSC-CM_Mat. Measurements include dominant frequency (DF), calcium transient durations (CaTD20, CaTD50, CaTD70, CaTD90), rise time, no background baseline fluorescence intensity (F0), normalized maximum fluorescence amplitude (Max ΔF/F0), inter-beat interval (IBI), and conduction velocity (CV). Data are presented as mean ± standard deviation, Flecainide-induced changes are shown for each condition.

**Table S4.** Statistical significance (p-values) for comparison of electrophysiological and calcium transient parameters across different culture conditions and rhythms. Conditions compared included 2D_Mat, hiPSC-CM_d11 and hiPSC-CM_Mat. Parameters evaluated include dominant frequency (DF), calcium transient durations (CaTD20, CaTD50, CaTD70, CaTD90, rise time, no background baseline fluorescence intensity (F0), normalized maximum fluorescence amplitude (Max ΔF/F0), inter-beat interval (IBI), and conduction velocity (CV). Significant differences (p<0.05) are highlighted in yellow. Statistical test include paired t-tests, and Pearson’s correlations, to evaluate differences between conditions and the impact of flecainide treatment.

| **Condition1** | **Condition2** | **Rhythm** | **Statistical Test** | **DF (Hz)** | **CaTD20 (ms)** | **CaTD50 (ms)** | **CaTD70 (ms)** | **CaTD90 (ms)** | **RiseTime (s)** | **F0 (a.u.)** | **Max ΔF/F0 (%)** | **IBI**  **(s)** | **CV (mm/s)** |
| --- | --- | --- | --- | --- | --- | --- | --- | --- | --- | --- | --- | --- | --- |
| **2D_Mat** | **hiPSC-CM_d11** | Basal | T-Test | 0,2375 | 0,0438 | 0,0320 | 0,0265 | 0,0325 | 0,0714 | 0,8903 | 0,9062 | 0,4832 | - |
| **2D_Mat** | **hiPSC-CM_Mat** | Basal | T-Test | 0,1171 | 0,3441 | 0,0819 | 0,0588 | 0,0221 | 0,8285 | 0,9471 | 0,2169 | 0,0150 | - |
| **2D_Mat** | **hiPSC-CM_d11** | Flecainide | T-Test | - | - | - | - | - | - | - | - | - | - |
| **2D_Mat** | **hiPSC-CM_Mat** | Flecainide | T-Test | 0,4364 | 0,8105 | 0,3932 | 0,2451 | 0,2289 | 0,0129 | 0,1677 | 0,9107 | 0,1074 | - |
| **2D_Mat** | **hiPSC-CM_d11** | 1Hz | T-Test | 0,0121 | 0,0153 | 0,0053 | 0,0010 | 0,0000 | 0,0071 | 0,0348 | 0,0151 | 0,0087 | - |
| **2D_Mat** | **hiPSC-CM_Mat** | 1Hz | T-Test | 0,4128 | 0,5911 | 0,1515 | 0,2287 | 0,3467 | 0,5381 | 0,8077 | 0,1903 | 0,1189 | - |
| **2D_Mat** | **hiPSC-CM_d11** | 2Hz | T-Test | 0,2420 | 0,8534 | 0,9235 | 0,9286 | 0,6868 | 0,0077 | - | - | - | - |
| **2D_Mat** | **hiPSC-CM_Mat** | 2Hz | T-Test | 0,0022 | 0,0238 | 0,0057 | 0,0077 | 0,0141 | 0,0143 | 0,6783 | 0,3110 | 0,1184 | - |
| **2D_Mat** | **hiPSC-CM_d11** | 3Hz | T-Test | 0,3564 | 0,2646 | 0,2849 | 0,3674 | 0,7909 | 0,0542 | 0,9235 | 0,0453 | 0,1843 | - |
| **2D_Mat** | **hiPSC-CM_Mat** | 3Hz | T-Test | 0,2239 | 0,6131 | 0,3756 | 0,3590 | 0,3595 | 0,9807 | 0,8020 | 0,0110 | 0,1927 | - |
| **hiPSC-CM_d11** | **hiPSC-CM_Mat** | Basal | T-Test | 0,0016 | 0,0001 | 0,0000 | 0,0000 | 0,0000 | 0,4469 | 0,9097 | 0,1760 | 0,0003 | - |
| **hiPSC-CM_d11** | **hiPSC-CM_Mat** | Flecainide | T-Test | - | - | - | - | - | - | - | - | - | - |
| **hiPSC-CM_d11** | **hiPSC-CM_Mat** | 1Hz | T-Test | 0,4537 | 0,0221 | 0,0053 | 0,0093 | 0,0711 | 0,0006 | 0,1304 | 0,0001 | 0,3707 | - |
| **hiPSC-CM_d11** | **hiPSC-CM_Mat** | 2Hz | T-Test | 0,9651 | 0,1769 | 0,0693 | 0,0795 | 0,1816 | 0,0330 | - | - | - | - |
| **hiPSC-CM_d11** | **hiPSC-CM_Mat** | 3Hz | T-Test | 0,5876 | 0,0498 | 0,0284 | 0,0484 | 0,2234 | 0,0926 | 0,9440 | 0,0000 | 0,6044 | - |
| **2D_Mat** | **-** | Basal vs Flecainide | T-Test | 0,1410 | 0,0503 | 0,0321 | 0,0280 | 0,0320 | 0,0083 | 0,9698 | 0,4488 | 0,1591 | 0,0591 |
| **hiPSC-CM_d11** | **-** | Basal vs Flecainide | T-Test | - | - | - | - | - | - | - | - | - | - |
| **hiPSC-CM_Mat** | **-** | Basal vs Flecainide | T-Test | 0,0058 | 0,0005 | 0,0000 | 0,0000 | 0,0000 | 0,0000 | 0,1221 | 0,6047 | 0,0004 | - |
| **2D_Mat** | **-** | 1-3Hz | ANOVA | 0,0507 | 0,1460 | 0,1207 | 0,0917 | 0,0691 |  | 0,1388 | 0,2192 | 0,1674 | 0,4339 |
| **hiPSC-CM_d11** | **-** | 1-3Hz | ANOVA | - | - | - | - | - |  | - | - | - | - |
| **hiPSC-CM_Mat** | **-** | 1-3Hz | ANOVA | 0,0019 | 0,0457 | 0,0174 | 0,0055 | 0,0008 |  | 0,6810 | 0,0710 | 0,0002 | - |
| **2D_Mat** | **-** | 0-3Hz | Pearsons | 0,0005 | 0,0110 | 0,0078 | 0,0048 | 0,0023 | 0,0293 | 0,0960 | 0,0813 | 0,0225 | 0,7866 |
| **hiPSC-CM_d11** | **-** | 0-3Hz | Pearsons | 0,1583 | 0,2780 | 0,2491 | 0,1804 | 0,1042 | 0,1548 | 0,7446 | 0,5216 | 0,6654 | - |
| **hiPSC-CM_Mat** | **-** | 0-3Hz | Pearsons | 0,0009 | 0,0751 | 0,0429 | 0,0172 | 0,0029 | 0,6301 | 0,3014 | 0,0582 | 0,0003 | - |

**Table S5. Designation, identifier, origin and source of each hiPSC line.**

| **hiPSCs** | **Identifier** | **Origin** | **Source** |
| --- | --- | --- | --- |
| hiPSC.1 | CBiPS1sv-4F-40 (RRID: CVCL V189) | Cord blood | Spanish Stem Cell Bank |
| hiPSC.2 | IMR90-4 | Fetal lung | WiCell |
| hiPSC.3 | DF19-9-11T.H | Foreskin fibroblasts | WiCell |

**Table S6. Coating, culture medium and CHIR concentration used at day 0 of CM differentiation for each of the hiPSC lines.**

| **hiPSC line** | **Plate**  **Coating** | **hiPSC Culture**  **Medium** | **CHIR concentration (µM)** |
| --- | --- | --- | --- |
| hiPSC.1 | Matrigel^®^Growth Factor Reduced (Corning) | TeSR-E8^TM^ (STEMCELL Technologies) | 7 |
| hiPSC.2 | Matrigel^®^ hESC-  Qualified Matrix (Corning) | mTeSR^TM^1 (STEMCELL Technologies) | 10 |
| hiPSC.3 | Matrigel^®^ hESC-Qualified Matrix (Corning) | mTeSR^TM^1 (STEMCELL Technologies) | 6.5 |

**Table S7. Antibodies for flow cytometry.**

| **Antibody** | **Antibody Type** | **Reference** | **Dilution** | **Epitope** |
| --- | --- | --- | --- | --- |
| Anti-SSEA-4, FITC | Primary conjugated | 560126 (BD Biosciences) | 1:9 | Extracellular |
| Isotype control, IgM, FITC | Primary conjugated | 553474 (BD Biosciences) | 1:400 | Extracellular |
| Anti-SSEA-1, FITC | Primary conjugated | 560127 (BD Biosciences) | 1:9 | Extracellular |
| Isotype control, IgM | Primary unconjugated | sc-3881 (Santa Cruz Biotechnology) | 1:7 | Extracellular |
| Anti-TRA-1-60 | Primary unconjugated | sc-21705 (Santa Cruz Biotechnology) | 1:7 | Extracellular |
| Isotype control, IgG | Primary unconjugated | EPR25A (Abcam) | 1:1000 | Intracellular |
| Anti-cTnT | Primary unconjugated | EPR20266 (Abcam) | 1:500 | Intracellular |
| Isotype control, IgG1 | Primary unconjugated | sc-3877 (Santa Cruz Biotechnology) | 1:2 | Intracellular |
| Anti-α-actinin | Primary unconjugated | A-7811 (Sigma Aldrich) | 1:500 | Intracellular |
| Alexa Fluor^TM^ 488,  anti-mouse IgM | Secondary | A-21042 (Invitrogen) | 1:200 |  |
| Alexa Fluor^TM^ 488,  anti-rabbit IgG1 | Secondary | A-11008 (Invitrogen) | 1:200 |  |
| Alexa Fluor^TM^ 488,  anti-mouse, IgG1 | Secondary | A-11001 (Invitrogen) | 1:200 |  |
| Alexa Fluor^TM^ 594,  anti-mouse, IgG1 | Secondary | A-11005 (Life Technologies) | 1:200 |  |

**Table S8. Antibodies for immunofluorescence.**

| **Antibody** | **Antibody Type** | **Reference** | **Dilution** |
| --- | --- | --- | --- |
| Anti-cTnT | Primary unconjugated | MA5-12960 (Invitrogen) | 1:100 |
| Anti-α-actinin | Primary unconjugated | A-7811 (Sigma Aldrich) | 1:100 |
| Anti-Ki-67 | Primary unconjugated | ab16667 (Abcam) | 1:100 |
| Anti-Vimentin | Primary unconjugated | ab16700 (Abcam) | 1:100 |
| Anti-Cx43 | Primary unconjugated | C6219 (Sigma Aldrich) | 1:100 |
| Alexa Fluor^TM^ 488,  anti-rabbit IgG1 | Secondary | A-11008 (Invitrogen) | 1:200 |
| Alexa Fluor^TM^ 594,  anti-mouse, IgG1 | Secondary | A-11005 (Life Technologies) | 1:200 |

**Table S9. List of primers used in RT-qPCR.**

| **Gene** | **Reference** |
| --- | --- |
| ***RPLP0*** | Hs99999902_m1 |
| ***GAPDH*** | Hs99999905_m1 |
| ***NANOG*** | Hs02387400_g1 |
| ***POU5F1*** | Hs00999632_g1 |
| [***NKX2-5***](https://www.ncbi.nlm.nih.gov/gene/1482) | Hs00231763_m1 |
| [***GATA4***](https://www.ncbi.nlm.nih.gov/gene/2626) | Hs00171403_m1 |
| [***TNNT2***](https://www.ncbi.nlm.nih.gov/gene/7139) | Hs00165960_m1 |
| [***TNNI1***](https://www.ncbi.nlm.nih.gov/gene/7135) | Hs00913333_m1 |
| [***TNNI3***](https://www.ncbi.nlm.nih.gov/gene/7137) | Hs00165957_m1 |
| [***MYH6***](https://www.ncbi.nlm.nih.gov/gene/4624) | Hs01101425_m1 |
| [***MYH7***](https://www.ncbi.nlm.nih.gov/gene/4625) | Hs01110632_m1 |
| [***MYL2***](https://www.ncbi.nlm.nih.gov/gene/4633) | Hs00166405_m1 |
| [***MYL7***](https://www.ncbi.nlm.nih.gov/gene/58498) | Hs00221909_m1 |

**Movie S1.**

hiPSC-CM after cardiac differentiation.

**Movie S2.**

hiPSC-CM after 11 days of expansion in 2D_Static.

**Movie S3.**

hiPSC-CM after 11 days of expansion in STB.

**Movie S4.**

hiPSC-CM after 11 days of expansion in STB_Agg.

**Movie S5.**

hiPSC-CM after 11 days of expansion in STB_10%O2.

**Movie S6.**

hiPSC-CM after 11 days of expansion in STB_10%O2_2L.

**Movie S7.**

Calcium transient optical mapping signal for 2D_Mat.

**Movie S8.**

Calcium transient optical mapping signal for hiPSC-CM_Mat.

**Supporting References**

[1] J. I. Laughner, F. S. Ng, M. S. Sulkin, R. M. Arthur, I. R. Efimov, *American Journal of Physiology-Heart and Circulatory Physiology* **2012**, *303*, H753.

[2] C. O’Shea, A. P. Holmes, T. Y. Yu, J. Winter, S. P. Wells, B. A. Parker, D. Fobian, D. M. Johnson, J. N. Correia, P. Kirchhof, L. Fabritz, K. Rajpoot, D. Pavlovic, *J Vis Exp* **2019**, *148*.

[3] J. L. Dynes, A. V Yeromin, M. D. Cahalan, *Journal of General Physiology* **2020**, *152*, e201812239.

[4] G. R. Ríos-Muñoz, Á. Arenal, A. Artés-Rodríguez, *Front Physiol* **2018**, *9*.
